# Exploring the medicinally important secondary metabolites landscape through the lens of transcriptome data in fenugreek (*Trigonella foenum graecum* L.)

**DOI:** 10.1038/s41598-022-17779-8

**Published:** 2022-08-08

**Authors:** Mahantesha B. N. Naika, Nitish Sathyanarayanan, Radha Sivarajan Sajeevan, Teerna Bhattacharyya, Pritha Ghosh, Meenakshi S. Iyer, Mahita Jarjapu, Adwait G. Joshi, K. Harini, K. Mohamed Shafi, Neha Kalmankar, Snehal D. Karpe, Bhavika Mam, Shaik Naseer Pasha, Ramanathan Sowdhamini

**Affiliations:** 1 National Centre for Biological Sciences (TIFR), GKVK Campus, Bengaluru, India; 2 Department of Biotechnology and Crop Improvement, K.R.C. College of Horticulture, University of Horticultural Sciences-Bagalkot, Arabhavi, Karnataka 591218 India; 3 The University of Trans-Disciplinary Health Sciences & Technology (TDU), Yelahanka, Bengaluru, Karnataka 560064 India

**Keywords:** Genome informatics, Phylogeny, Protein function predictions, Proteome informatics, Sequence annotation, Secondary metabolism, Transcriptomics, Bioinformatics, Reverse transcription polymerase chain reaction, RNA sequencing, Next-generation sequencing

## Abstract

Fenugreek (*Trigonella foenum-graecum* L.) is a self-pollinated leguminous crop belonging to the Fabaceae family. It is a multipurpose crop used as herb, spice, vegetable and forage. It is a traditional medicinal plant in India attributed with several nutritional and medicinal properties including antidiabetic and anticancer. We have performed a combined transcriptome assembly from RNA sequencing data derived from leaf, stem and root tissues. Around 209,831 transcripts were deciphered from the assembly of 92% completeness and an N50 of 1382 bases. Whilst secondary metabolites of medicinal value, such as trigonelline, diosgenin, 4-hydroxyisoleucine and quercetin, are distributed in several tissues, we report transcripts that bear sequence signatures of enzymes involved in the biosynthesis of such metabolites and are highly expressed in leaves, stem and roots. One of the antidiabetic alkaloid, trigonelline and its biosynthesising enzyme, is highly abundant in leaves. These findings are of value to nutritional and the pharmaceutical industry.

## Introduction

Fenugreek (*Trigonella foenum-graecum* L.) is a dicotyledonous leguminous self-pollinated crop with chromosome number 2*n* = 16 belonging to the Fabaceae (Leguminosae) family^1^. It is native to India and eastern Mediterranean countries. The genus *Trigonella* includes 140 species distributed globally, among which 107 species occur in Asia^2^. In India mainly two types are commonly cultivated i.e., common fenugreek (*Trigonella foenum-graecum* L.) for leaves, stem and seeds, and Kasuri fenugreek (*Trigonella corniculata* L.) for leaves. Fenugreek is one of the oldest known medicinal plants of India and is traditionally used in ayurvedic medicines. It is considered to be a multipurpose crop for its food and non-food uses such as herb, spice, fodder, and its nutraceutical, pharmaceutical and therapeutic properties^3^. In 2017, Venkata et al.^4^ reviewed fenugreek for its broad pharmaceutical usage on preclinical and clinical research on human diseases and antipathogenic properties.

**Figure 1:**
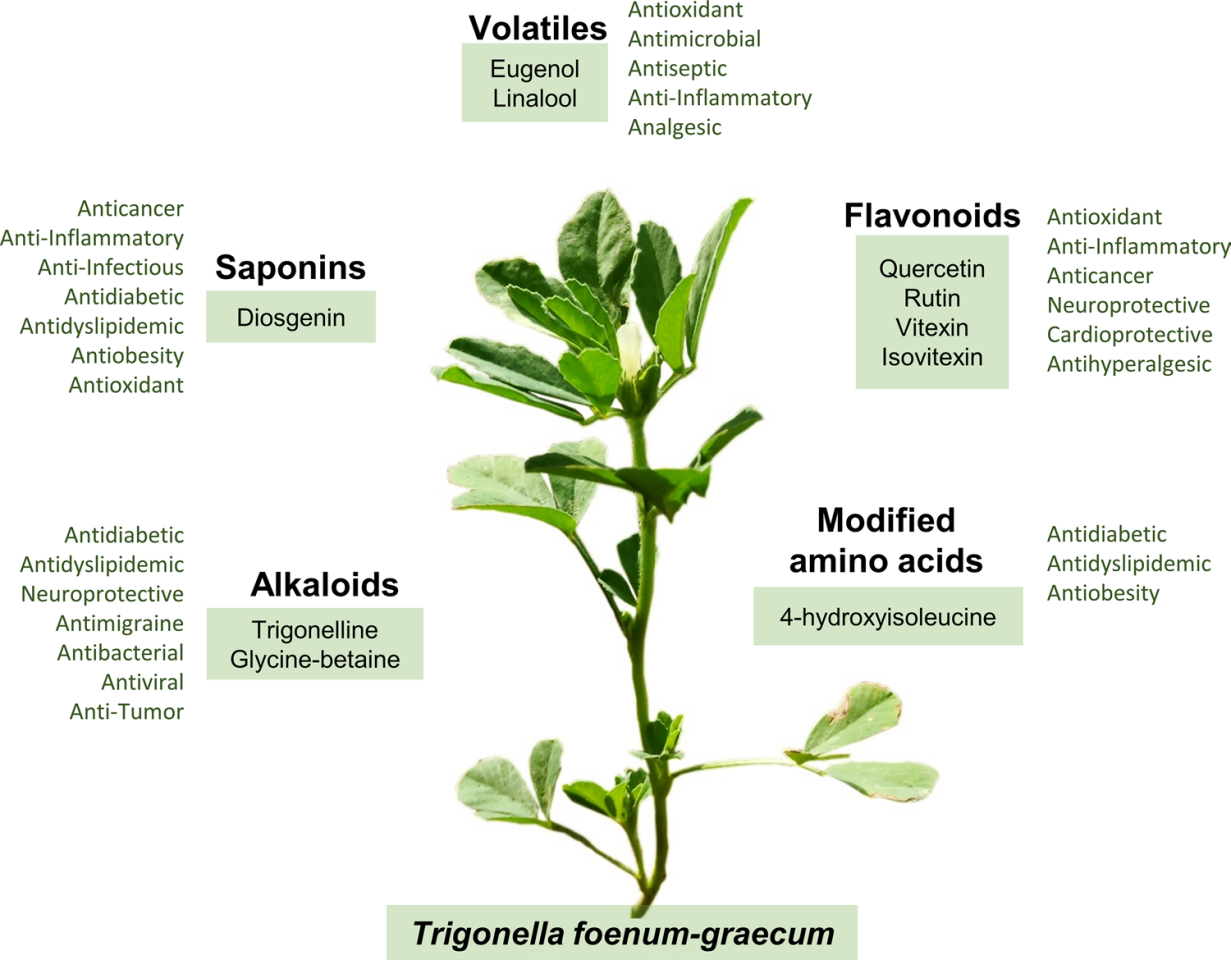
A snapshot of selected secondary metabolites produced by *Trigonella foenum-graecum* (shown in image is the AFG-1 variety). The abundance of these compounds make fenugreek a repertoire of nutraceutical and pharmaceutical properties.

Although there has been considerable advancement in the field of drug development, medicinal plants are being increasingly utilised for treating various diseases owing to their therapeutic properties and safety. Fenugreek has a high content of dietary fibre, vitamin and mineral content as well as bioactive compounds such as modified amino acids, phenolic acids, alkaloids and saponins (Fig. 1). It has multiple health benefits, such as antidiabetic, anticancer, galactagogic, helps in digestion, hepatoprotective effects, regulatory functions, antioxidant properties, and works against anorexia, antilithogenic, hepatoprotective effect, antipathogenic properties and several other medicinal properties^5–7^. It is also Generally Recognized As Safe (GRAS) while using as a flavour by the U.S. Food and Drug Administration (FDA)^8^.

A recent review reports the presence of more than 100 phytochemicals in the fenugreek seeds^9^. Among the several important secondary metabolites produced by fenugreek, diosgenin, trigonelline and 4-hydroxyisoleucine are three compounds which have been implicated in the anti-diabetic properties of the plant (Fig. 1). Diosgenin, a steroidal saponin, is an important starting material for the preparation of several steroidal drugs in the pharmaceutical industry and has shown high potential in the treatment of various types of disorders such as cancer, hypercholesterolemia, inflammation, and several types of infections^10^. The pharmacological activities of trigonelline, an alkaloid, include hypoglycaemic, hypolipidemic, neuroprotective, antimigraine, sedative, memory-improving, antibacterial, antiviral, and anti-tumour activities^11^. 4-hydroxyisoleucine (4-HIL), a modified amino acid is another bioactive compound abundant in fenugreek seeds. The antidiabetic property of 4-HIL is attributed to its ability to induce insulin secretion in a glucose-dependent manner^12,13^. There are several other important classes of metabolites such as volatiles (eugenol, linalool), flavonoids (quercetin, rutin, vitexin and isovitexin) which render fenugreek multiple medicinal properties^7,14^.

There have been a limited number of studies for identification of potential genes involved in biosynthesis of bioactive compounds attributing the nutritional and pharmaceutical properties of the fenugreek as well as differential expression of these genes across the tissues of the plant. The transcriptome studies in fenugreek will pave the way for identification of candidate genes involved in medicinal as well as environmental stress tolerance properties of the plant and these can be taken up further in molecular breeding programs^15,16^. Transcriptomics provide the details of genes associated with functions at the cellular and tissue level; analysis of transcriptomic data from different tissues of the organism is particularly useful in understanding differential gene expression^17^. Towards this goal, we have performed transcriptome sequencing from leaf, stem and root tissues of fenugreek and estimated the transcript abundance across these tissues. We use this data to explore the secondary metabolite landscape of the plant by deciphering genes involved in synthesis of several nutritionally and medicinally important metabolites. We have also quantified the expression of selected genes and performed quantification of the selected metabolites.

## Materials and methods

### Sequencing and assembly of the transcriptome

Seeds of *T. foenum-graecum* (Ajmer Fenugreek-1 (AFG-1) variety) were procured from College of Horticulture, GKVK Campus, Bengaluru with due permission. All the studies on *T. foenum-graecum* were carried out in accordance with relevant institutional, national and international guidelines and legislation. For transcriptome sequencing, RNA from three different tissues-leaf, stem and root, was isolated from one month old plants using the Plant Spectrum Plant RNA extraction kit (Sigma-Aldrich). These tissue samples, with two biological replicates, were sequenced using Illumina HiSeq 1000 platform with 100 base pairs (bp) read length. Trimmomatic was used to process the reads using default settings^18^. A total of 226.5 million reads were obtained after quality processing from six libraries. These reads were assembled de novo using Trinity with default parameters^19^. BUSCO was used to assess the completeness of the transcriptome assembly^20^.

### Transcriptome functional annotation

TransDecoder (v3.0.0) was used to identify the candidate coding regions within transcript sequences obtained from Trinity^19^. A length cut-off of 100 bases was used to predict Open Reading Frames (ORFs). Function assignment for amino acid sequences was obtained using BLASTP^21^ against UniProt Viridiplantae database with an E-value cut-off of 10^–5^^22^ and domain identification was performed using hmmscan of the HMMER suite (v.3.1b2)^23^ against Pfam (v.31.0) using an E-value cut-off of 10^–5^^24^. For transcripts, BLASTX^21^ was performed against the above sequence database with the default parameters. Additional annotation was rendered using SignalP (v.4.0)^25^, TMHMM (v.2.0)^26^ and RNAmmer (v.1.2)^27^ to extract information on signal peptides, transmembrane regions and RNA sequences. Gene ontology (GO) terms^28^, orthologous relationships (eggNOG)^29^ and pathway information (KEGG)^30^ were also mined.

### Detection of orthologous relationships

Orthology analysis of *T. foenum-graecum* was carried out with the well-studied model plants *Arabidopsis thaliana* and *Oryza sativa* and plants which belong to the same tribe as fenugreek (Trifolieae) viz. *Trifolium pratense* and *Medicago truncatula* using OrthoMCL^31^. An E-value cut-off of 10^–5^ was used for the all-against-all BLAST runs. Additionally, 34 different plant species proteomes from Phytozome (v.12)^32^ were used for large scale orthology analysis using Proteinortho (v5.11)^33^ (Supplementary Table 1) with an E-value cut-off of 10^–5^.

The concatenated nucleotide sequence alignments for ribulose-1,5-bisphosphate carboxylase/oxygenase large subunit (rbcL), maturase K (matK) and internal transcribed spacer 1 and 2 (ITS1 and ITS2) were used for constructing species phylogenetic tree for the species belonging to subclass Rosids. In case of partial sequences, the alignment was trimmed. The individual sequences were aligned using MAFFT^34^ with maximum allowance for aligning gapped regions and the separate alignments were concatenated. Phylogenetic reconstruction was performed using the Neighbour-Joining method implemented in MAFFT with 100 bootstraps. The orthologue distribution obtained from Proteinortho analysis along with their phylogenetic tree for comparison amongst these closely related species was plotted. Through orthogroup analysis, the in-paralogous groups and singletons of *T. foenum-graecum* common to both methods were identified. The sequences were compared with each other with respect to their expression profiles in different tissues, Pfam domains^22^ and GO terms^28^.

### Transcript abundance estimation

The reads from the three tissues were mapped onto the de novo transcriptome assembly using Bowtie2 (v2.3.0). The expression counts were obtained using eXpress (v1.5.1) and RSEM^35–37^. Transcripts per Million (TPM) values were calculated for all the transcripts. The average TPM value was considered for the biological replicates and compared across the three tissues.

### Identification and analysis of genes involved in synthesis of secondary metabolites

The genes involved in synthesis of secondary metabolites belonging to alkaloids, saponins, volatiles and flavonoids, present in *T. foenum-graecum* were identified using strategy adapted from Joshi et al.^38^. The pathway information and related functional annotation was extracted from multiple sources such as PlantCyc MetaCyc, KEGG and literature^11,30,39–42^. The pathway images were adapted from PlantCyc or literature. The sequences for the enzymes involved in the synthesis of secondary metabolites serving as start points for sequence search were identified from UniProt^22^. This was followed with creation and curation of multiple sequence alignment profiles including functionally important residues (FIR), typically used for selecting trusted homologues^21,43^. Such profiles were implemented to search the transcriptome data for potential hits and were ascertained by presence of the FIRs. The phylogenetic trees were constructed using the neighbor-joining method from MEGA (v10)^44^ for 1000 bootstraps, to assess co-clustering of the candidate genes with the curated start points. The phylogenetic trees obtained were visualized and edited in Figtree (v1.4.2)^45^ and iToL^46^.

### Expression analysis of genes involved in production of important secondary metabolites

The genes identified to be involved in production of secondary metabolites trigonelline and diosgenin were validated using quantitative reverse transcription PCR (qRT-PCR). The details of methods used for RNA isolation, qRT-PCR quantification are described in the Supplementary Text (Sect. 1.1).

### Quantification of diosgenin, trigonelline and 4-hydroxyisoleucine in different tissue samples

The quantification of diosgenin, trigonelline and 4-hydroxyisoleucine was carried out from leaf, stem, root, and seed tissue samples using HLPC-PDA method. The details of this method are described in the methods part of Supplementary Text (Sect. 1.2).

## Results

We carried out transcriptome sequencing of three tissues (leaf, stem and root) of fenugreek plant and constructed a combined assembly along with functional annotation. We investigated the orthology relationship with other plant species and also estimated the transcript abundance across tissues. Further, we explored the secondary metabolite landscape of fenugreek considering metabolites with reported medicinal properties from several classes of compounds (alkaloids, saponins, volatiles, flavonoids, etc.) and mined for the enzymes involved in their synthesis.

### Transcriptome assembly and functional annotation

Combined transcriptome assembly was obtained from three different tissues (leaf, stem and root) with N50 of 1382 bases. The 169.5 Mb assembly contained 209,831 transcripts. This assembly was 92.2% complete (95.49% using partial core genes) as assessed through the BUSCO^20^.

The candidate coding regions within the fenugreek transcriptome were predicted using Transdecoder program. A set of 64,670 ORFs (complete: 37,294, partial: 27,376—terminally partial: 18,743, internally partial: 8633) were predicted from the assembly which varied from 200 to 2000 base pairs. We obtained annotations, using BLASTX against UniProt Viridiplantae database, for 26% of transcripts (Table 1). Whereas, among the predicted ORFs, we obtained annotation for 90% of ORFs, through BLASTP using E-value threshold of 10^–5^ and 50% query coverage cut-off. Most of these ORFs (76%) were associated with *Medicago truncatula*, a closely related species, as the best hit.

**Table 1:** Transcriptome assembly and annotation statistics.

Details of the transcriptome assembly						
Assembly	Total number of transcripts	Number of transcripts > 1 Kb	N50 (bp)	Size (Mb)	GC%	BUSCO completeness
Combined assembly	209,831	48,685	1382	169.56	36.58	92.2% (95.49% including partial genes)
**Functional annotation of transcripts and predicted amino acid sequences**						
**Annotation of transcripts (209,831)**						
BLASTX	54,577 (26%)					
GO	60,798 (30%)					
rRNAs (RNAmmer)	8 (0.04%)					
**Annotation of predicted amino acid sequences (Total ORFs: 64,670): complete: 37,294, partial: 27,376 (terminally: 18,743, internally: 8633)**						
Sequence hits (BLASTP)	58,504 (90%)					
Protein domains (Pfam-hmmscan)	42,524 (66%)					
Signal peptides (SignalP v.4.0)	3743 (6%)					
Transmembrane helices (TMHMM v.2.0)	12,417 (19%)					
Orthologous groups (eggNOG/COG)	37,243 (58%)					
Association with KEGG Pathway	38,930 (60%)					
GO terms	38,790 (59%)					

Among the predicted ORFs, 66% (42,524) could be associated with Pfam domains, representing 4400 unique Pfam domains. The leucine-rich repeat (LRR), pentatricopeptide repeat (PPR), tetratricopeptide repeats (TPR) and several transcription factor binding (WD40, F-box, AAA) domain families were the most abundant annotated domains observed. Similar to other angiosperms, several other Pfam domains such as, protein serine/threonine kinases, protein tyrosine kinases, calcium binding EF-hand and ankyrin repeats were found in abundance (Supplementary Fig. 1A). We could associate GO terms to more than 50% of total ORFs. The top enriched GO terms were classified into molecular function (MF), biological process (BP) and cellular component (CC) (Supplementary Fig. 1B). The enriched terms under MF included transporter activity, transcription regulator, antioxidant activity and nutrient reservoir. Whereas, some of the most enriched BP terms were metabolic process, response to stimuli and immune system process. It was observed that transmembrane helices were present in 19% of ORFs, signal peptides were predicted in 6% of ORFs, whereas eight rRNAs were identified. Of the total number of amino acid sequences obtained from the transcriptome assembly, 59% were mapped to functional annotation databases, namely eggNOG (orthogroups), GO and KEGG pathways (Table 1; complete annotation data available in Supplementary Data 1).

### Detection of orthologous relationships

**Figure 2:**
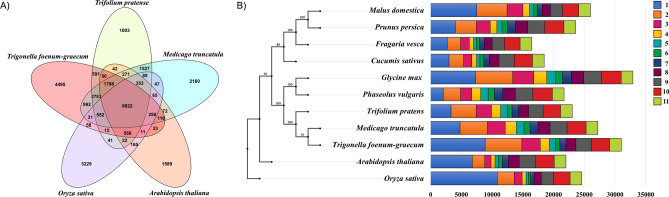
Orthology analyses (**A**) OrthoMCL: Venn diagram representing the common and unique orthogroups for the five proteomes studied. (**B**) Proteinortho results showing the distribution of orthogroups between few selected plants from the subclass Rosids (including *Arabidopsis thaliana* and few plants from Faboideae and Rosaceae) and *Oryza sativa*. Each coloured section represents the number of species with which the orthogroups are shared.

The orthologs were detected using OrthoMCL and Proteinortho for fenugreek and closely related plants (Fig. 2A,B and Supplementary Fig. 2). Among the five plants used for OrthoMCL analysis, 8822 orthogroups were common to all whereas, for Proteinortho, 114 orthogroups were common across 39 plants considered for the analysis. It was observed that the orthogroup distribution was highly similar across all the plants from subfamily Faboideae (*G. max, P. vulgaris, T. pratense, M. truncatula* and *T. foenum-graecum*). We constructed the phylogenetic tree using selected plants from subclass Rosids and *O. sativa*. (Fig. 2B).

We observed 20,230 fenugreek specific paragroups and singletons, among which only the longest ORFs (15,712, 24% of all predicted gene products) were assessed for their functional annotation (hereafter referred to as singletons). A total of 6300 of the singletons in fenugreek proteins show presence of 1771 unique Pfam families. PPR, LRR, protein kinases, protein tyrosine kinases, zinc knuckle and reverse transcriptase domain were among the most abundant Pfam domains in the singleton proteins (Supplementary Fig. 3A). Almost half of the fenugreek singletons (7235) were found to have Gene Ontology (GO) annotation. Nucleotide/nucleic acid binding, metal ion binding were abundant in molecular functions within GO terms (Supplementary Fig. 3B). Translation, regulation of transcription and defence response were the most abundant biological process (Supplementary Fig. 3C). The most common cellular component term within these GO terms was ‘integral component of membrane’ (Supplementary Fig. 3D).

### Transcript abundance estimation

**Figure 3:**
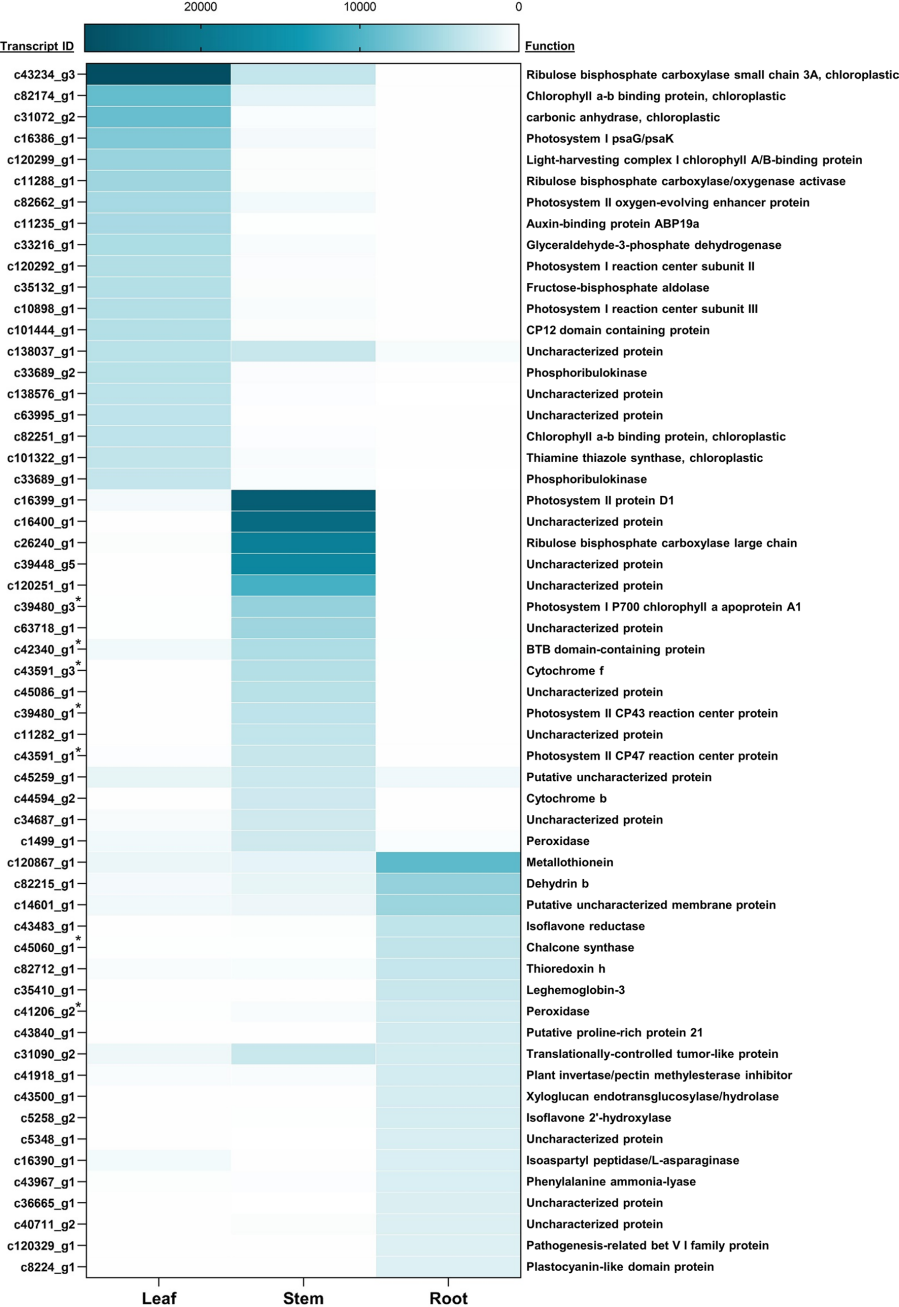
Heatmap for top 20 most abundant transcripts (based on average TPM values) from leaf, stem and root tissues. The * sign indicates transcripts with multiple annotations. The annotation derived from the best hit have been ascribed for such transcripts.

The transcript abundance across all three tissues (leaf, stem and root) was assessed. For each tissue, the average TPM value between the replicates was considered for each transcript. The top 20 highly abundant transcripts were identified from each tissue and are represented as a heatmap (Fig. 3). While observing variation between highly abundant transcripts in each tissue, we could identify six transcripts (protease inhibitor, metallothionein, *dehydrin B*, *thioredoxin H*, plant invertase inhibitor and translationally controlled tumor-like protein), which were highly expressed across three tissues. Among the top most abundant transcripts in the leaf sample, transcripts mostly related to photosynthesis, for example, ribulose bisphosphate carboxylase, chlorophyll a–b binding protein, carbonic anhydrase etc., were observed. In the stem, apart from photosynthesis related transcripts, few cytochrome genes and pathogenesis related protein families were among the most abundant transcripts. In the root, the top three highly abundant annotated transcripts included metallothionein, *dehydrin B* and *Nmr-A* like family of genes. These genes are well documented to be involved in drought tolerance in crops^47–50^. The tissue-wide average TPM values for enzymes involved in synthesis of several secondary metabolites considered in this work are mentioned in the subsequent sections and documented in the Supplementary Table 3.

### Identification and analysis of genes involved in synthesis of secondary metabolites

We explored the secondary metabolites landscape in fenugreek with an aim to identify the transcripts involved in their biosynthesis. The candidate metabolites and the enzymes involved in their biosynthesis were chosen following an extensive literature survey. These spanned several classes of secondary metabolites: alkaloids, saponins, volatiles and flavonoids.

#### Alkaloids

Alkaloids are heterocyclic nitrogen containing compounds displaying a wide array of pharmacological properties. These compounds are not only associated with plant growth and defence but are a storage source of nitrogen^51^. We considered trigonelline and glycine-betaine from the alkaloid class found in fenugreek. We pursued identification of enzymes involved in their biosynthesis by adopting the strategy described in Joshi et al.^38^.

**Figure 4:**
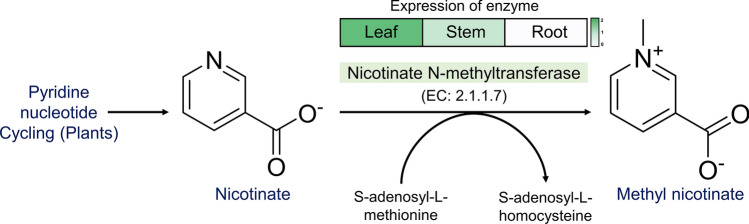
Trigonelline biosynthesis pathway and relative abundance across tissues of the transcript encoding the enzyme-Nicotinate *N*-methyltransferase (represented as heatmap of average TPM value in log2 scale, in green-white colour scale). The pathway has been adapted from Plant Metabolic Network (PMN), https://pmn.plantcyc.org/PLANT/NEW-IMAGE?type=PATHWAY&object=PWY-5110&detail-level=3, on www.plantcyc.org, Aug 12, 2021.

Trigonelline is a derivative of nicotinic acid, first isolated from fenugreek (*Trigonella foenum-graecum*). Trigonelline has several medicinal properties such as hypoglycaemic, neuroprotective, antibacterial, and anti-tumour activities^52^. It is synthesised through purine nucleotide cycling, wherein, nicotinate is methylated to methyl nicotinate (trigonelline) by nicotinate N-methyltransferase (NNMT) (EC: 2.1.1.7). We selected and curated protein sequences for this enzyme found in other plants, as a start point to carry out sequence search in fenugreek transcriptome. There are five functionally important residues (FIRs), a substrate-binding motif Asn 21, Tyr 120, His 124 a catalytic motif Thr 264 and a SAM binding motif in the NNMT enzyme^42^. Although 12 hits were obtained, a single hit (Tfoe_c19814_g2_i1_m7872) was identified based on co-clustering with *Medicago truncatula* sequence (NCBI RefSeq ID: XP_013464471.1) (Supplementary Fig. 4E) with a sequence identity of 92.6% and a 100% query coverage. Further this hit was validated through mapping of the FIRs (Supplementary Fig. 4A–D). Based on the average TPM values, this transcript was most abundant in the leaf tissue sample (Fig. 4, Supplementary Table 3). This trend was also similar to the expression level estimation through qRT-PCR (Supplementary Fig. 5A). Trigonelline, as a compound, was quantified across different tissues of fenugreek (described further in “Quantification of metabolites in different tissue samples”).

Glycine betaine is one of the major osmoprotectants in plants and also contributes to maintaining membrane and cell stability. It plays a protective role against inflammation, carcinogenesis and neurodegenerative diseases^53–55^. It was identified in the extract of seeds of fenugreek^56^. It is synthesized in plants by the oxidation of choline through a two-step process (Supplementary Data 2). The first step is catalyzed by choline monooxygenase (CHMO) (EC: 1.14.15.7) which is an unusual iron-sulfur containing enzyme. We identified one hit (Tfoe_c38951_g1_i2_m30577) from fenugreek which contained both the motifs (Rieske 2Fe-2S and non-heme binding region). The second step catalyzes the formation of glycine-betaine from betaine-aldehyde by betaine aldehyde dehydrogenase (BADH) (EC: 1.2.1.8)^57^. We identified one hit for BADH (Tfoe_c82300_g1_i1_m72031) and mapped the FIRs (proton acceptor: Glu260, Nucleophile: Cys294 and cation-pi interacting Trp285, Trp456). The hits for both the enzymes in fenugreek co-clustered with the *M. truncatula* proteins (Supplementary Data 2). Both these transcripts were relatively abundant in the leaf tissue sample (Supplementary Table 3).

#### Saponins

Saponins are amphipathic glycosides grouped phenomenologically by the soap-like foaming they produce when shaken in aqueous solutions. These metabolites are found in the plant families-Sapindaceae, Aceraceae, Hippocastanaceae and Fabaceae^58^. We focussed on the biosynthetic pathway of diosgenin, a steroidal sapogenin attributed to treatment of metabolic disorders such as diabetes and hypercholesterolemia and also implicated in treatment of cancer, inflammation and several infections^10^. Tissue-wide diosgenin content varies in fenugreek plant and is reported to be present in highest quantity in seeds. Moreover, its quantity differs across several cultivars and is considered one of the criterion in crop improvement programs^59^.

From the proposed biosynthetic pathway of diosgenin, as per Ciura et al.^60^, (Supplementary Data 2), putative sequences corresponding to the last two enzymes (sterol-3β-glucosyltransferase and β-glucosidase) were identified from the set of protein sequences obtained from the transcriptome assembly. Sterol-3β-glucosyltransferase (EC: 2.4.1.173) contains two motifs-putative steroid binding domain (PSBD) motif and plant secondary product glucosyltransferase (PSPG) motif^61^. The hits obtained from the searches against the proteins sequences were filtered for the presence of these two motifs and of the four hits that contained this motif, from the FIR analysis, two transcripts (Tfoe_c26467_g1_i1_m12030 and Tfoe_c33087_g1_i3_m19456) expressing significantly in all three tissues of the plant were considered as true hits (referred henceforth as sterol-3β-glucosyltransferase-1 and -2). While sterol-3β-glucosyltransferase-1 (Tfoe_c26467_g1_i1_m12030) clustered with a sequence from a closely related plant, *M. truncatula*, the sterol-3β-glucosyltransferase-2 (Tfoe_c33087_g1_i3_m19456) clustered with the enzyme sequence from *Glycine soja*. Sterol-3β-glucosyltransferase-1 expressed well in all tissues of the plant (leaf, stem and root) while sterol-3β-glucosyltransferase-2 showed relatively higher average TPM value in leaves and this was further validated using qRT-PCR (Supplementary Fig. 5B,C, Supplementary Table 3).

The last enzyme in the biosynthetic pathway of diosgenin, β-glucosidase (EC: 3.2.1.21), is known to contain two catalytic glutamate residues which are part of the (I/V)TENG and TFNEP motifs and these have been identified from crystal structures of β-glucosidase from rice in apo and bound forms (2RGL and 2RGM)^62^. We identified the β-glucosidase in fenugreek (Tfoe_c120410_g1_i1_m78951) and confirmed the presence of these motifs. Although, we found a total of 22 such hits which had > 70% query coverage and > 30% sequence identity, with the query β-glucosidase sequences from Viridiplantae, the selected candidate protein Tfoe_c120410_g1_i1_m78951 clustered with the enzyme from *M. truncatula* and was thus considered a true hit. This transcript showed high average TPM value in root followed by stem and the same relative expression pattern was observed from qRT-PCR validation (Supplementary Fig. 5D, Supplementary Table 3). The metabolic pathway, sequence motif, phylogenetic tree and the expression data for all diosgenin synthesising enzymes is documented in Supplementary Data 2.

#### Volatiles

The volatiles are a class of compounds which typically include aromatic organic compounds. These compounds are used in perfumes, flavours, topical formulations, etc. The medicinal properties of essential volatiles have been known since ancient times. We have explored the enzymes involved in the biosynthesis of eugenol and linalool which are volatile oils found in fenugreek^63,64^. The therapeutic potential of eugenol and isoeugenol, which are phenolic compounds, is attributed to antioxidant potency and anti-inflammatory benefits^65,66^. Linalool, a terpene, has been reported to show anticancer, analgesic, anxiolytic and other neuroprotective properties^67^. Eugenol and isoeugenol are phenylpropene volatiles that have a phenyl ring with a propenyl side chain synthesized from coniferyl alcohol precursor in multiple enzymatic reactions (Supplementary Data 2). We studied three enzymes in the eugenol synthesis pathway: Coniferyl alcohol acetyltransferase, eugenol synthase and isoeugenol synthase^68^. Coniferyl alcohol acetyltransferase (EC: 2.3.1.224) associated with two proteins (Tfoe_c37673_g1_i1_m27189, Tfoe_c102741_g1_i1_m76275), however, Tfoe_c37673_g1_i1_m27189 was selected to be the true candidate gene (marked in Supplementary Data 2) since it co-clustered with the *M. truncatula* protein and was also supported by the presence of the FIR. Similarly, the candidate proteins from fenugreek for eugenol synthase (EC: 1.1.1.318) and the isoeugenol synthase (EC: 1.1.1.319) co-clustered with clades belonging to known enzymes (highlighted in Supplementary Data 2). The average TPM values across tissues for each of the enzymes involved in synthesis of eugenol are represented in Supplementary Table 3. The transcripts corresponding to both the enzymes were found to be most abundant in root compared to the other tissues.

In the linalool synthesis pathway we studied three enzymes: Farnesyl-pyrophosphate synthase, (3S)-linalool synthase and (3R)-linalool synthase^69,70^. (3S)-linalool and (3R)-linalool are two enantiomeric forms of naturally occurring monoterpenoids. The candidate protein from fenugreek for farnesyl-pyrophosphate synthase (EC: 2.5.1.1) (Tfoe_c5299_g1_i1_m1972) co-clustered with another legume *Lupinus albus* (Fabaceae family). The (3S)-linalool synthase (EC: 4.2.3.25) (Tfoe_c11093_g1_i1_m4195, Tfoe_c11093_g1_i2_m4196) and (3R)-linalool synthase (EC: 4.2.3.26) (Tfoe_c44084_g4_i2_m55446) co-clustered with other sequence homologues (as highlighted in the Supplementary Data 2). The average TPM values for each of the enzymes involved in synthesis of linalool are represented in Supplementary Table 3. While the first enzyme-farnesyl-pyrophosphate synthase, is expressed highest in root compared to the other tissues, (3S)-linalool synthase and (3R)-linalool synthase is expressed highly in leaves.

#### Flavonoids

Flavonoids, an important class of secondary metabolites with a polyphenolic structure, widely found in fruits, vegetables and certain beverages. This class of compounds has been linked to a variety of nutraceutical, pharmaceutical, and medicinal properties, including antioxidative, anti-inflammatory, antimutagenic, and anticarcinogenic properties^71^. We studied biosynthetic pathways of four flavonoids (quercetin, vitexin, isovitexin and rutin) known to be present in fenugreek^63^. These metabolites are synthesised from coumaroyl-CoA through different enzymatic reactions. The flavonoids share a common flavonoid backbone structure with differences in their hydroxylation patterns.

Quercetin biosynthesis pathway involves six enzymes [4-Coumarate-CoA ligase (EC: 6.2.1.12), chalcone synthase (EC: 2.3.1.74), chalcone flavone isomerase (EC: 5.5.1.6), flavanone 3-hydroxylase (EC: 1.14.11.9)/flavonol synthase (EC: 1.14.20.6), tricin synthase (EC: 2.1.1.175) and flavonoid 3′-monooxygenase (EC: 1.14.14.82)] that convert coumaroyl-CoA to quercetin through several intermediates^17^. For each of the six enzymes involved in the pathway, multiple transcript hits were identified in fenugreek as mentioned in Supplementary Table 3. The sequence hits for these enzymes were validated as true hits, based on co-clustering with well annotated sequence of same sub-family observed in the phylogenetic tree (as described in Methods “Identification and analysis of genes involved in synthesis of secondary metabolites”). It was observed that the selected enzymes are highly abundant in roots of the plant followed by the stem (Supplementary Table 3, Supplementary Data 2).

We further studied biosynthetic pathways for vitexin and isovitexin, which are two enantiomeric metabolites. The enzymes involved their pathway are identical^72^, and they produce these metabolites in a mixture. A three-step reaction is known to be involved in the conversion of naringenin to vitexin/isovitexin. The first two reactions are enzymatic while the third step (hydration reaction)^73^, although, has been hypothesized to be an enzymatic one, there is no definitive enzyme that has been isolated for it. For the first two enzymes [naringenin 2-hydroxylase (EC: 1.14.14.162) and C-glucosyltransferase (EC: 2.4.1.360)] known in the reaction, four and 13 hits were obtained from the fenugreek transcriptome Supplementary Table 3. These hits were validated using phylogenetic analysis of the enzymes from the same sub-family (Supplementary Data 2). The highest abundance of the naringenin 2-hydroxylase in the pathway was observed in the stem, while for C-glucosyltransferase, although there were 13 hits identified fenugreek, most of them were highly expressed in leaf.

The third flavonoid, rutin, is synthesised in plants from taxol. The pathway involves two enzymatic reactions. While vitexin/isovitexin are obtained by C-glycosylation, rutin is obtained by O-glycosylation of the substrates. The enzymes involved in the pathway are flavonol-3-o-glucosyltransferase (EC: 2.4.1.91) and flavonol-3-o-glucoside rhamnosyltransferase (EC: 2.4.1.15)^74^. There were 11 hits identified for flavonol-3-o-glucosyltransferase, and most of them had higher expression value in leaf and root tissue. In case of flavonol-3-o-glucoside rhamnosyltransferase a single hit (Tfoe_c121323_g1_i1_m79650) was identified with a high abundance in leaf (Supplementary Table 3, Supplementary Data 2).

### Quantification of metabolites in different tissue samples

Fenugreek is a traditionally well-known plant for its medicinal properties and the presence of complex mixtures of key phytochemicals renders this plant such benefits^4,63^. In the literature, details of these chemical constituents in fenugreek tissue extracts have mainly been demonstrated by bio-analytical techniques. We quantified, presence of important metabolites- diosgenin, trigonelline and 4-hydroxyisoleucine (4-HIL) in different tissues (leaf, stem, root and seed) of fenugreek, by HPLC and mass spectrometry. Although high-quality RNA could not be purified from the seed tissue, we used it nonetheless for metabolite quantification. Diosgenin and trigonelline were quantified using HPLC–PDA and 4-HIL was quantified using LC–MS analysis from extracts of different tissues as described in Supplementary Methods Sect. 1.2. Supplementary Fig. 6A,B show the standard curve of diosgenin and trigonelline respectively*.* The concentration of each compound was quantitatively determined by comparison of the peak area of the standard with that of the samples. Supplementary Fig. 7A–D,E–H refers to the HPLC peaks which showed the presence of trigonelline and diosgenin, and Supplementary Fig. 7L–N refers to LC profile from LC–MS data of 4-HIL. As seen in Supplementary Table 4, seed gave the highest concentration of diosgenin i.e. 1.56 µg/mg of the tissue followed by the root (1.036 µg/mg). Leaf tissue showed the highest concentration of trigonelline i.e. 4.715 µg/mg of the tissue followed by the seed (2.698 µg/mg). 4-HIL was detected at a very high concentration of 84.77 µg/mg in the dry seed and 77.48 µg/mg from the leaf. The concentration of 4-HIL was observed to significantly decrease with water-soaked seeds post 12 h and 24 h. The concentrations of these medicinally important metabolites vary across several cultivars of fenugreek^75,76^.

## Discussion

We have surveyed here the landscape of secondary metabolites in fenugreek, which are medicinally important, through the lens of transcriptome data. Apart from being a food crop of semi-arid nature, fenugreek is not only a food source but has been used in traditional medicines in several parts of the world. The medicinal properties of this drought tolerant leguminous plant can largely be attributed to an array of secondary metabolites produced in it^63^. In this study we have identified candidate genes involved in the biosynthesis of such metabolites belonging to four major classes, alkaloids, saponins, volatiles and flavonoids. The process adopted for identification of such genes in the transcriptome has been previously implemented in *Ocimum tenuiflorum*, *Moringa oleifera*^17,38,77^. It involved creation and curation of a knowledgebase for candidate gene products from other (closely) related plants and applying it through a rigorous sequence search and validation process to ascertain the transcripts in the fenugreek transcriptome assembly. The raw data from leaf, stem and root tissue samples was processed and assembled into a set of transcripts and their level of expression was compared across these tissues in measure of average TPM values. In particular, several drought responsive genes such as metallothionein, dehydrin B and Nmr-A like family of genes as described in the results “Transcript abundance estimation” were found to be among most abundant transcripts, especially in the root tissue sample.

While identifying candidate genes involved in biosynthesis of secondary metabolites, it is vital to select appropriate start points for the rigorous sequence search and subsequent validation. We rely on multiple sources such as PlantCyc, MetaCyc, KEGG pathway and metabolites cited in literature^11,39–42^ to create such a knowledge base of start points. It further helps in identification of FIRs which resolves selection of true candidate genes along with the co-clustering of proteins from fenugreek transcripts with the selected start points. For instance, we considered the trigonelline synthesising enzyme NNMT (“Alkaloids”, Fig. 4), which methylates nicotinate to methyl nicotinate or trigonelline using *S*-adenosyl-l-methionine (SAM) as the methyl donor. We identified 12 hits from the transcriptome through the sequence search, however, we make use of the knowledge-driven filtering to ascertain the correct annotation. We identified a single hit (Tfoe_c19814_g2_i1_m7872) in which we could find all the functionally important residues viz. the catalytic residue Thr 264, substrate-binding motif Asn 21, Tyr 120, His 124 and a SAM binding motif (Supplementary Fig. 4A–D). Subsequently this sequence co-clustered with the NNMT sequence (XP_013464471.1_MEDTR) from *Medicago truncatula*, which is closely related to fenugreek from the Trifolieae tribe (Supplementary Fig. 4E). These multiple checkpoints helped in selecting the correct fenugreek hit for rendering the annotation. The TPM values for this transcript were compared across all the tissues and found to be higher in leaves. This trend was followed in the qRT-PCR based expression (Supplementary Fig. 5A) as well the metabolite quantification (“Quantification of metabolites in different tissue samples”), suggesting that the fenugreek leaves are a rich source of this anti-diabetic compound, trigonelline. This knowledge-driven protocol was applied in selecting the correct start points and ascertaining the true fenugreek hits for all the other alkaloids, saponins, flavonoids and volatiles (described in the Results “Identification and analysis of genes involved in synthesis of secondary metabolites”). The candidate genes identified for the production of volatiles- eugenol, isoeugenol and linalool were found to be highly abundant in roots except for the enzyme (3S)-linalool synthase and (3R)-linalool synthase which were abundant in leaves compared to other tissues. The transcripts for the enzymes involved in the biosynthesis of the flavonoids-vitexin, isovitexin and rutin were found to be highly abundant in leaves, whereas, for quercetin biosynthesis the transcripts were abundant in the root and stem.

In the case of the diosgenin pathway, which is well studied in fenugreek^78^, there are two enzymes which were explored in the current study. Sterol-3β-glucosyltransferase (the penultimate enzyme) is associated with four hits in fenugreek. However, based on identification of PSBD and PSPG motifs and co-clustering with sequences from closely related leguminous plants, ensured identification of two true candidate genes (Tfoe_c26467_g1_i1_m12030, Tfoe_c33087_g1_i3_m19456) (“Saponins”). The first hit was found to be abundant across all the three tissues- leaves, stem and root while the second hit was found to be high in leaves. This was observed through the average TPM values across the tissue. Additionally, the qRT-PCR quantification also corroborated with this trend. The last enzyme in diosgenin biosynthesis, β-glucosidase, despite presence of (I/V)TENG and TFNEP motifs in all of the associated 22 hits, only a single hit was selected as the candidate gene based on co-clustering with *M. truncatula* protein sequence. This enzyme was found to be abundant in root and stem from the TPM values, which was corroborated by the qRT-PCR studies (“Saponins”). Diosgenin quantification from different tissues also indicated predominantly high concentrations in the root (“Quantification of metabolites in different tissue samples”). There are several transcriptome studies available in NCBI for fenugreek albeit with different cultivars and under different study conditions (BioProject IDs: PRJNA544308, PRJNA508420, PRJNA383660). We confirmed the presence of both the diosgenin pathway enzymes in their corresponding SRA datasets. We observed more than 98% sequence identity and close to 90% query coverage for the candidate genes from our study. This suggested that the candidate genes identified in our study agree well with the publicly available data for fenugreek.

Several fenugreek cultivars are widely cultivated across India. Traditionally, the AFG-1 variety, described in this study, has been one of the cultivars commonly grown across India, with bold seeds and good yield^79^. Many of the fenugreek cultivars grown across India face the powdery mildew disease, including the AFG-1 variety^80^. Ongoing work in UHS, Bagalkot, India (unpublished), on Indian fenugreek cultivars for identification of biotic stress resistant genotype, has observed that among selected cultivars, Kasuri methi showed high immune response towards the biotic stress arising from the powdery mildew disease. This could be further utilized to understand disease resistance mechanisms in fenugreek cultivars. The current transcriptome data will assist in establishing the genetic determinants for the nutritional and medicinal properties among the cultivars. It will also help us to estimate the important secondary metabolites and develop varieties with high nutraceutical value. This will further pave the way to develop and breed biotic and abiotic tolerant cultivars of fenugreek.

## Conclusions

We present here the transcriptome of the Ajmer Fenugreek-1 (AFG-1) variety of *T. foenum-graecum.* We identified candidate genes of enzymes involved in the biosynthesis of secondary metabolites such as alkaloids, saponins, flavonoids and volatile compounds. Some of these secondary metabolites are of immense value in the pharmaceutical and nutraceutical industries. Among these, diosgenin and trigonelline are known to be specific to fenugreek and are antidiabetic, antioxidant, anti-inflammatory, antiobesity along with several other medicinal values. The abundance of transcripts encoding these candidate genes was assessed and validated through qRT-PCR for tissue-level expression. Additionally, these metabolites were quantified across the tissues. Identification of genes involved in secondary metabolites biosynthesis in fenugreek will aid in bioengineering efforts for large scale production. Further, this transcriptome can be utilised for cross comparison of several cultivars and through molecular breeding programs lead to crop improvement.

## Supplementary Information

Supplementary Figure 1.

Supplementary Figure 2.

Supplementary Figure 3.

Supplementary Figure 4.

Supplementary Figure 5.

Supplementary Figure 6.

Supplementary Figure 7.

Supplementary Information.

## Data Availability

NCBI BioProject: PRJNA734905, BioSample: (leaf replicates: SAMN19548760, SAMN19548761; stem replicates: SAMN19548765, SAMN19548764; root replicates: SAMN19548762, SAMN19548763), SRA accession: (leaf replicates: SRR14721911, SRR14721912; stem replicates: SRR14721915, SRR14721916; root replicates: SRR14721913, SRR14721914). The Supplementary Data is available at the following link: http://caps.ncbs.res.in/download/tfoe_data/. The details are indexed in Supplementary Text file (“Materials and methods”).

## Abbreviations

Tfoe: *Trigonella foenum-graecum*
AFG-1: Ajmer Fenugreek-1
ORF: Open reading frame
TPM: Transcripts per million
FIR: Functionally important residues
qRT-PCR: Quantitative reverse transcriptase polymerase chain reaction
4-HIL: 4-Hydroxyleucine
GO: Gene ontology
NNMT: Nicotinate *N*-methyl transferase
BADH: Betaine aldehyde dehydrogenase
CHMO: Choline monooxygenase
PSBP: Putative sterol binding domain
PSPG: Plant secondary product glycosyltransferase
HPLC–PDA: High performance liquid chromatography–photo diode array
LC–MS: Liquid chromatography–mass spectrometry
