# Supplementary figures and images for: Exploring the medicinally important secondary metabolites landscape through the lens of transcriptome data in fenugreek (Trigonella foenum graecum L.)
Source: Sci Rep. 2022 Aug 8;12:13534. doi: 10.1038/s41598-022-17779-8 (PMC9359999; doi:10.1038/s41598-022-17779-8)

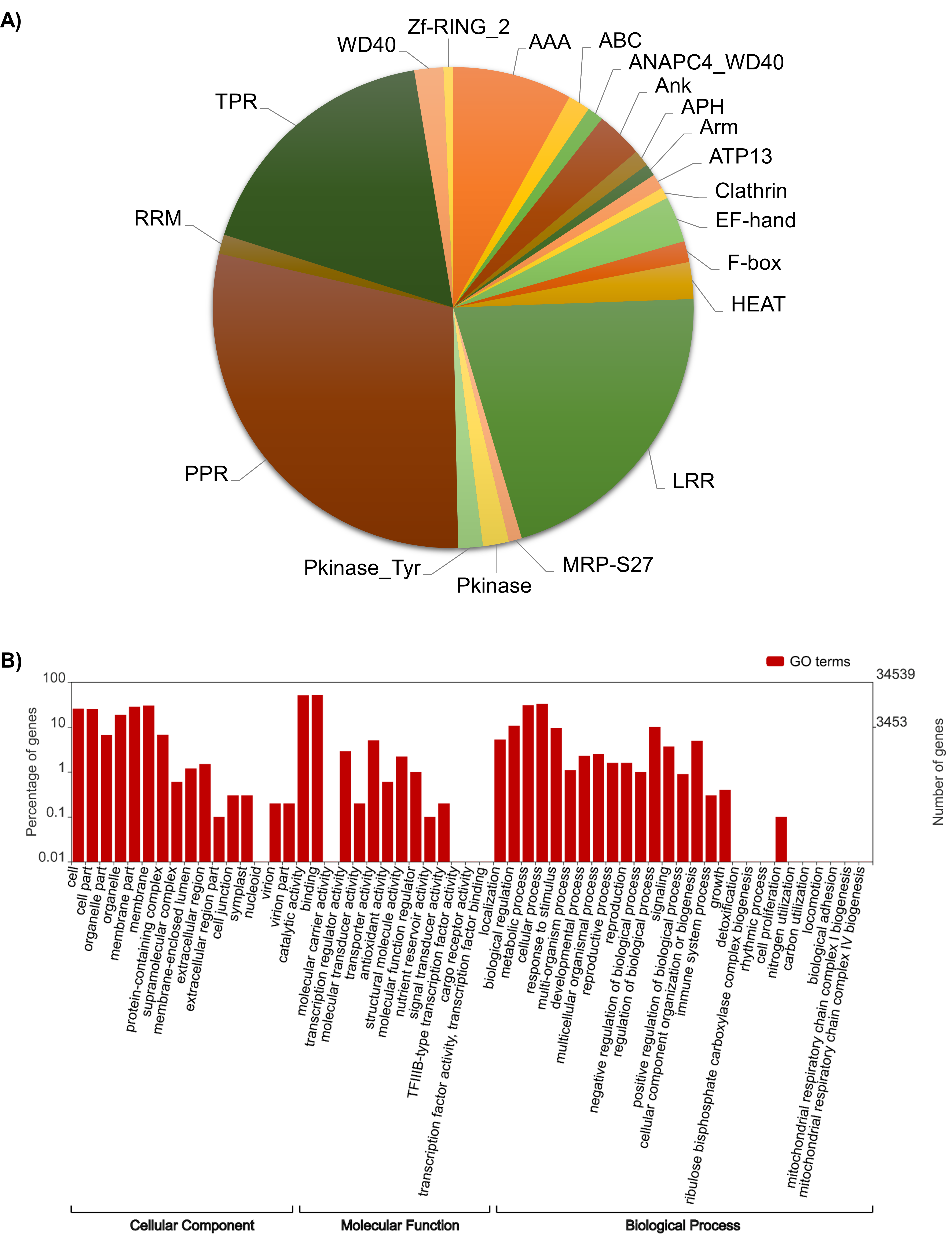

Supplement: Supplementary file 1 — Supplementary Figure 1. [file 41598_2022_17779_MOESM1_ESM.tif]

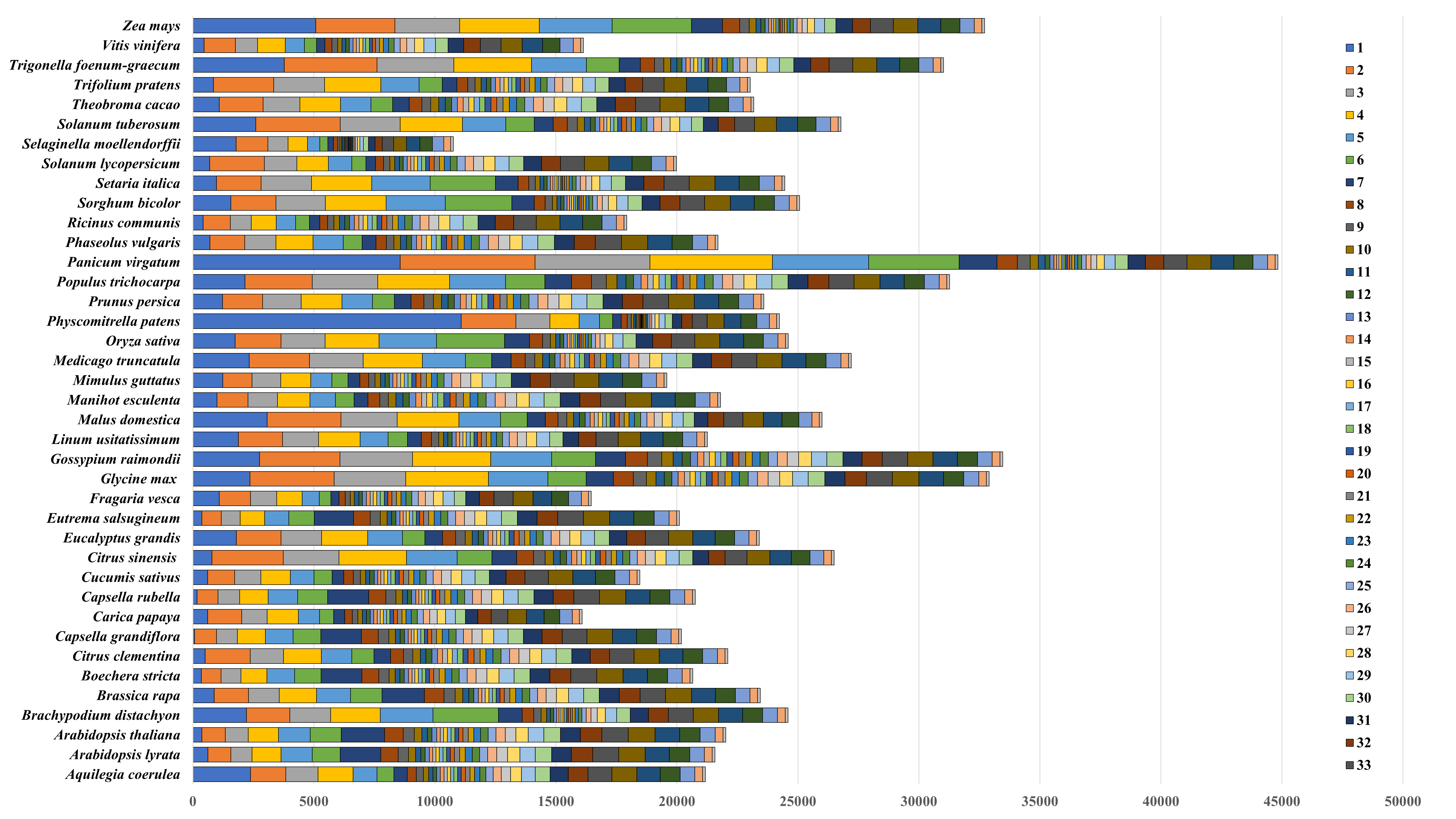

Supplement: Supplementary file 2 — Supplementary Figure 2. [file 41598_2022_17779_MOESM2_ESM.tif]

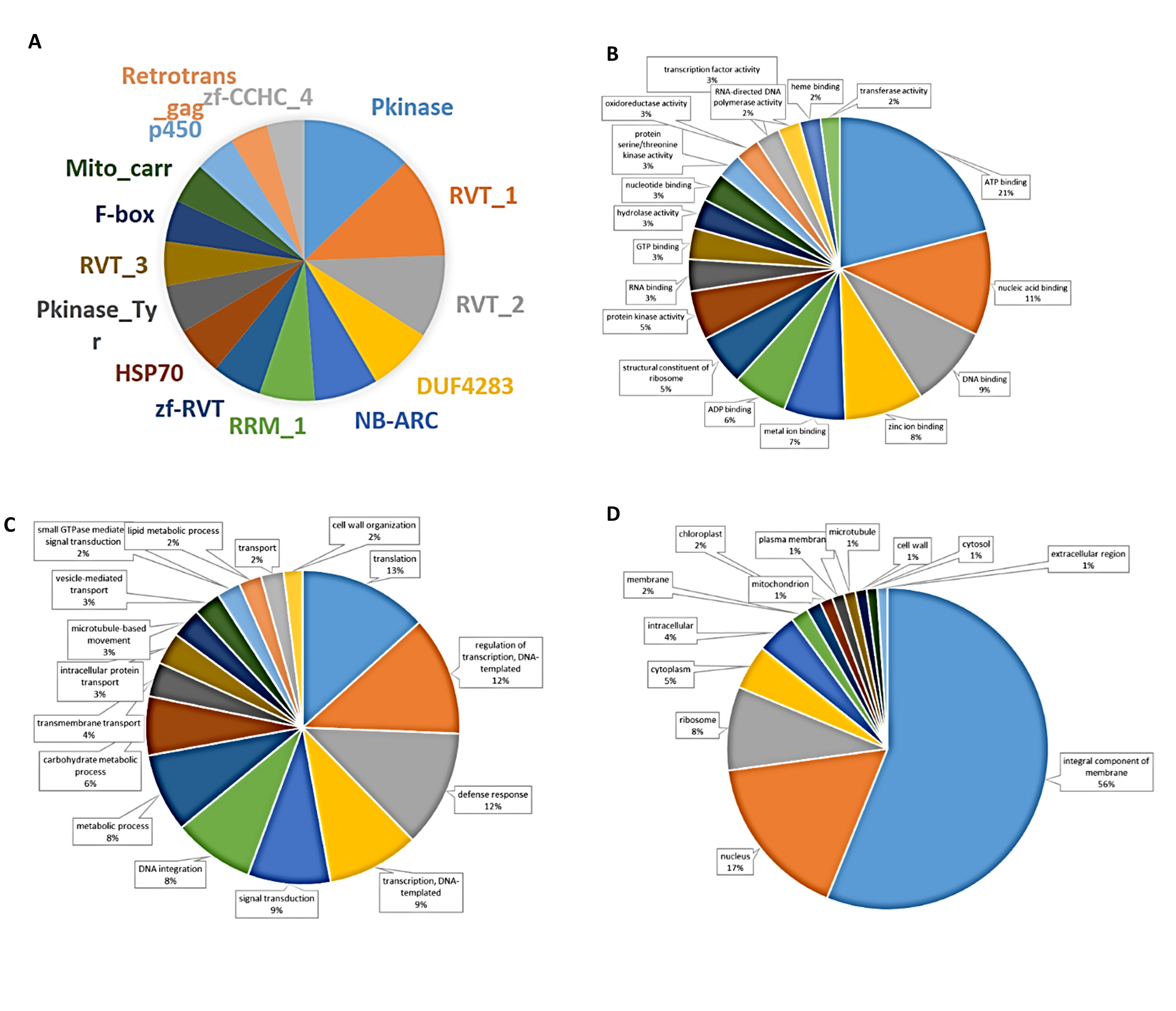

Supplement: Supplementary file 3 — Supplementary Figure 3. [file 41598_2022_17779_MOESM3_ESM.tif]

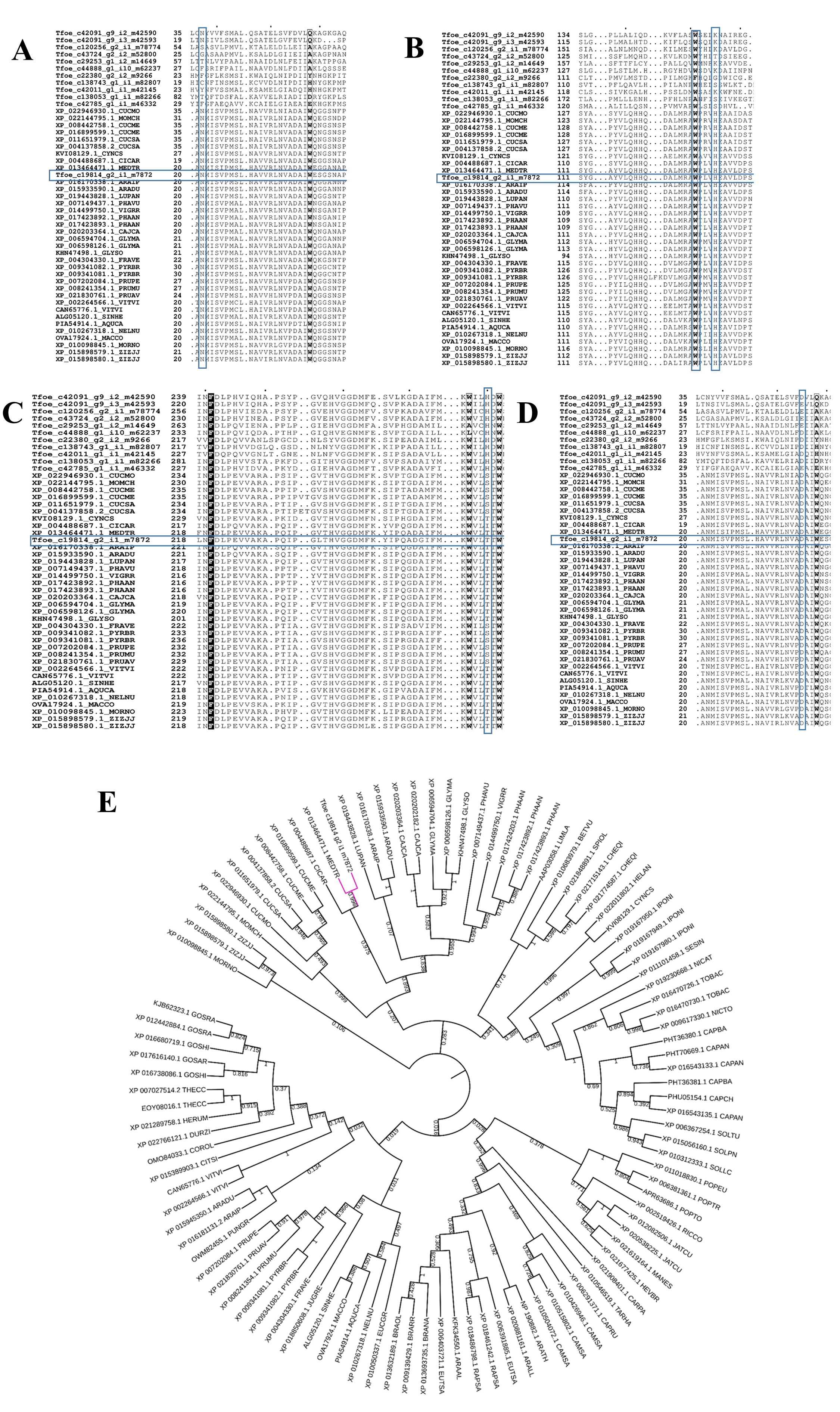

Supplement: Supplementary file 4 — Supplementary Figure 4. [file 41598_2022_17779_MOESM4_ESM.tif]

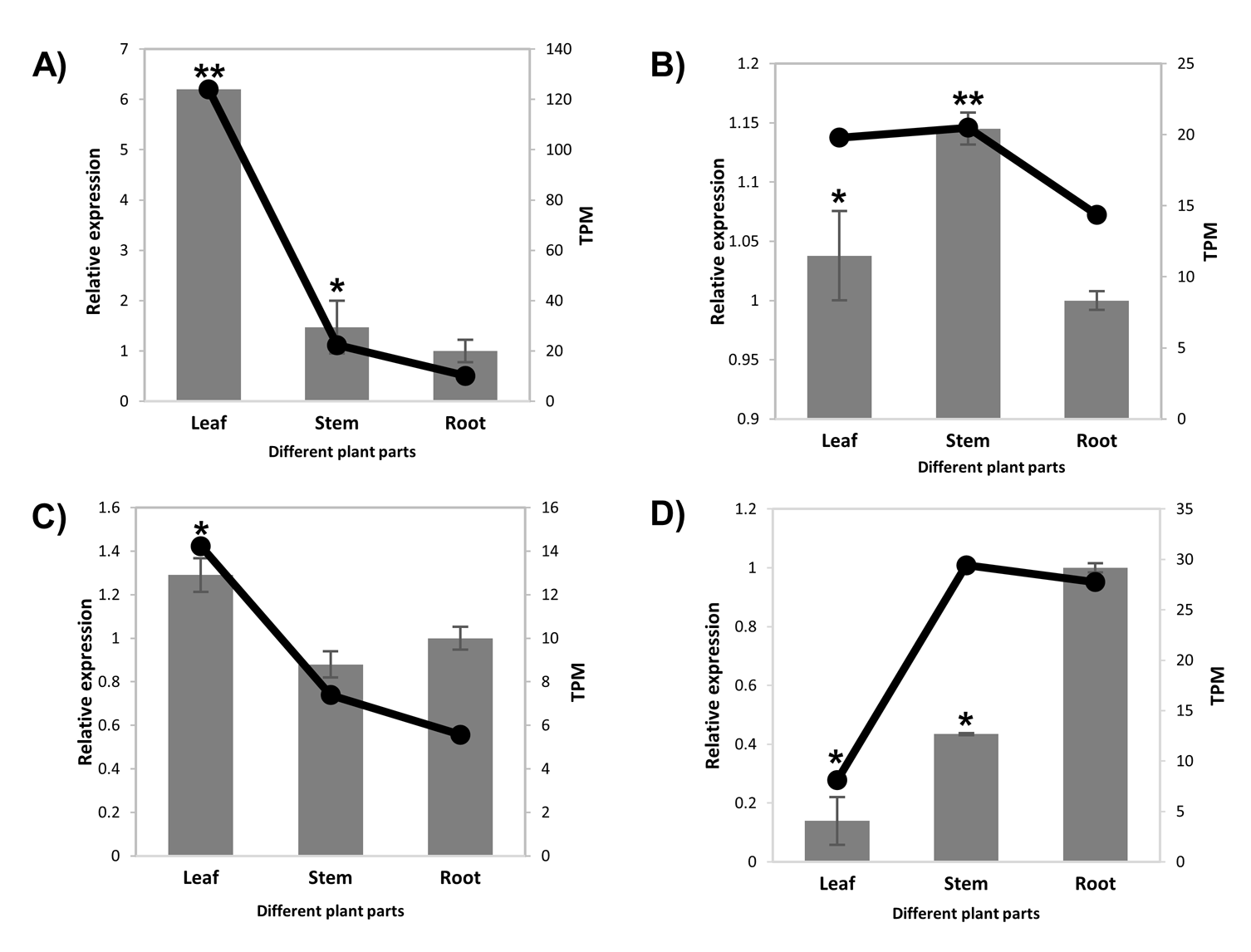

Supplement: Supplementary file 5 — Supplementary Figure 5. [file 41598_2022_17779_MOESM5_ESM.tif]

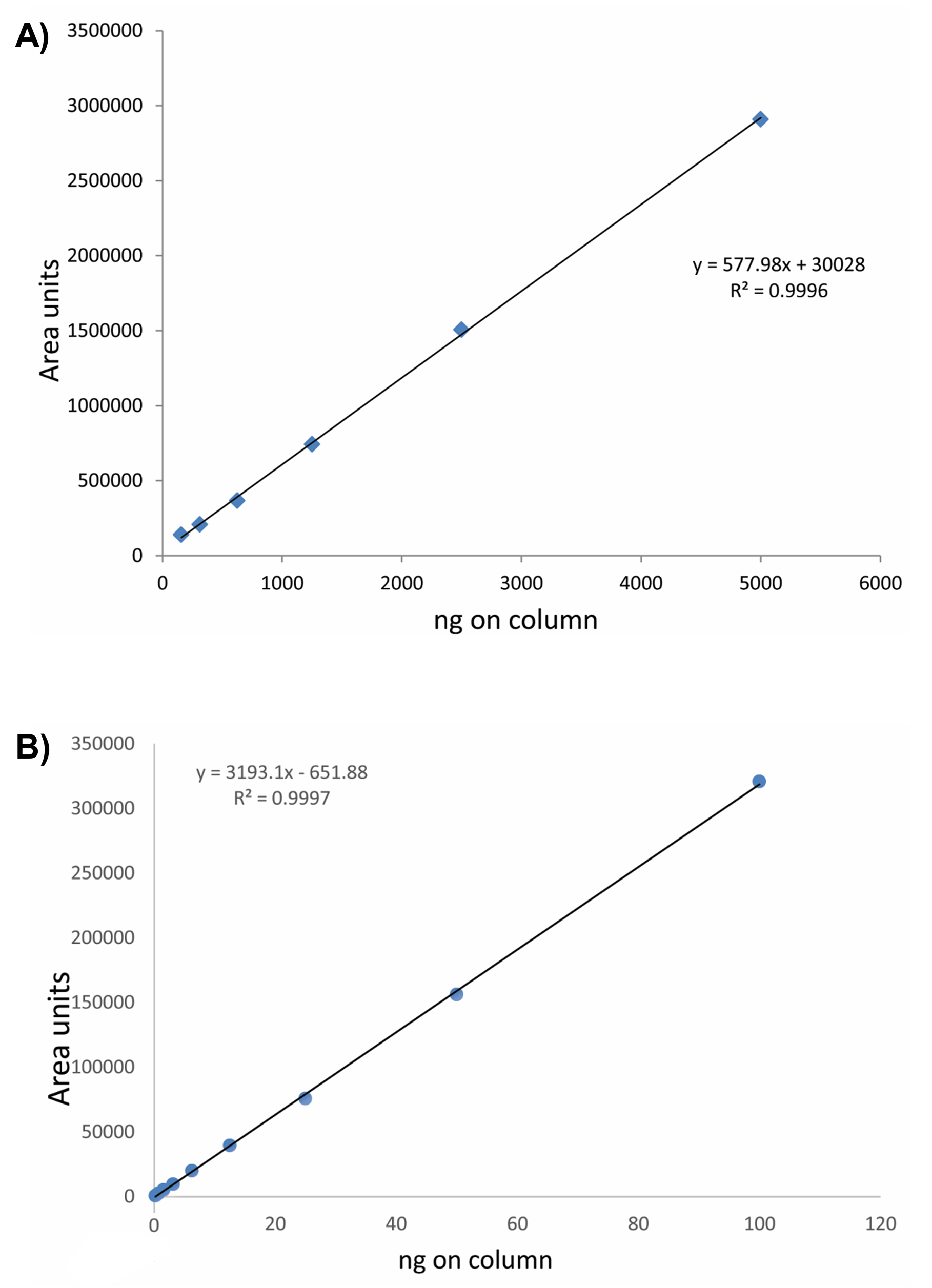

Supplement: Supplementary file 6 — Supplementary Figure 6. [file 41598_2022_17779_MOESM6_ESM.tif]

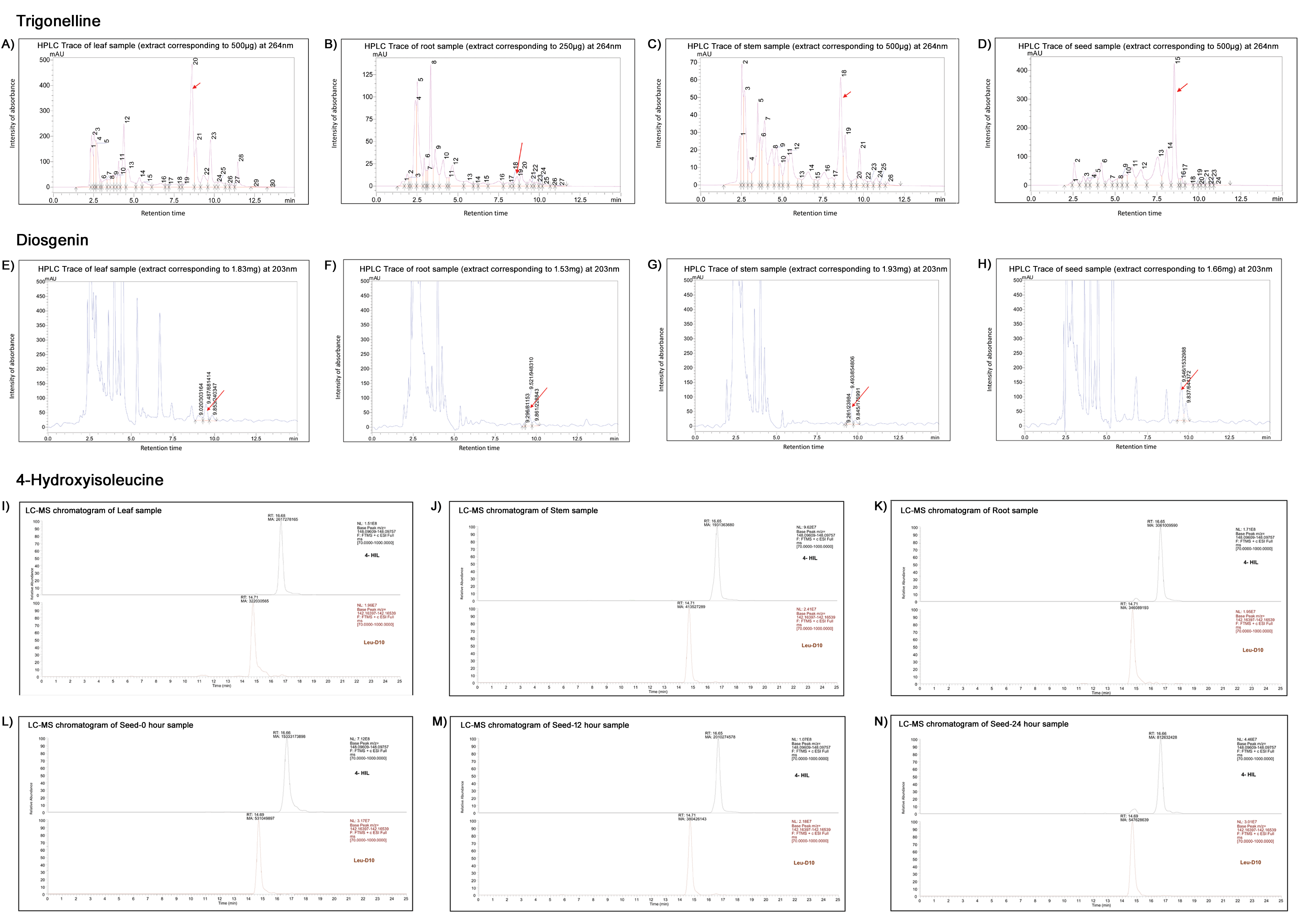

Supplement: Supplementary file 7 — Supplementary Figure 7. [file 41598_2022_17779_MOESM7_ESM.tif]
