# Supplementary material for: Exploring the medicinally important secondary metabolites landscape through the lens of transcriptome data in fenugreek (Trigonella foenum graecum L.)
Source: Sci Rep. 2022 Aug 8;12:13534. doi: 10.1038/s41598-022-17779-8 (PMC9359999; doi:10.1038/s41598-022-17779-8)
Supplement: Supplementary file 8 — Supplementary Information. [file 41598_2022_17779_MOESM8_ESM.docx]

**Supplementary file:**

**Exploring the medicinally important secondary metabolites landscape through the lens of transcriptome data in fenugreek (*Trigonella foenum graecum* L.)**

Mahantesha B.N. Naika^1,2^, Sathyanarayanan Nitish^1,3^, Radha Sivarajan Sajeevan^1^, Bhattacharyya Teerna^1^, Ghosh Pritha^1^, Iyer Meenakshi S.^1^, Jarjapu Mahita^1^, Joshi Adwait G.^1^, K. Harini^1^, K. Mohamed Shafi^1,3^, Kalmankar Neha^1,3^, Karpe Snehal D.^1^, Mam Bhavika^1,3^, Shaik Naseer Pasha^1^, Ramanathan Sowdhamini^1*^

^1^ National Centre for Biological Sciences (TIFR), GKVK campus, Bangalore, India

^2^ Department of Biotechnology and Crop Improvement, K.R.C. College of Horticulture, Arabhavi 591218, University of Horticultural Sciences-Bagalkot, Karnataka, India

^3^ The University of Trans-Disciplinary Health Sciences & Technology (TDU), Yelahanka, Bangalore 560064, Karnataka, India

**^*^ Corresponding author: mini@ncbs.res.in**

**1. Supplementary Methods:**

**1.1 Expression analysis of genes involved in production of important secondary metabolites**

We measured the expression of candidate transcripts involved in the production of selected secondary metabolites produced by fenugreek. Fenugreek cultivar AFG-1 seeds were soaked in distilled water overnight and later transferred to cheese cloth wetted with water for the radical protrusion. The germinated seeds were transferred to a plastic tray (30x22x7cm) filled with autoclaved soilrite (a mixture of horticulture grade perlite with Irish peat moss and exfoliated vermiculite supplied by Kel Perlite, Bangalore, India) moistened with one-fourth strength of MS macro salts. The plants were grown at ordinary day light conditions in a greenhouse with a relative humidity of 70% and a minimum and maximum temperatures of 22 and 26°C, respectively. The leaf, stem, and root samples were collected from one-month old plants and were immediately frozen in liquid nitrogen and stored at -80°C until further use. Total RNA was isolated using 100 mg of plant tissues using the Spectrum^TM^ Plant Total RNA Kit (Sigma-Aldrich) ^1^. RNA isolated was quantified using the nanodrop method and a total of 4μg total RNA was used for the complementary DNA (cDNA) preparation in a 20μl reaction volume using the SuperScript™ III First-Strand Synthesis SuperMix (Thermo Fisher Scientific) following the manufacturer's protocol after treating the total RNA with RNase-free Deoxyribonuclease I (DNase 1, 1U, Thermo Fisher Scientific). The quantitative real-time PCR (qRT-PCR) was performed using a CFX96 qRT-PCR detection system (Bio-Rad, Hercules, CA, USA) in triplicates in a final reaction volume of 20μl containing gene-specific forward and reverse primers (10 pmol/μL each), cDNA (2μl), and 10μl of 2× iQ SYBR green super mix (Bio-Rad, California, USA). The reaction conditions were as follows - initial denaturation of 95°C for 3 min, followed by 35 cycles - denaturation at 95°C for 15s, annealing at 60°C for 20s, and extension of 72°C for 20s. The melt curve analysis was performed with 95°C denaturation for 1 min, annealing at 50°C for 1 min, and stepwise denaturation from 50 to 95°C with 0.5°C increase and 20s hold. The 2^−∆∆Ct^ method ^2,3^ was used to calculate the relative expression level of each gene using the internal reference gene glyceraldehyde 3-phosphate dehydrogenase (*GAPDH*). Three biological replicates were averaged and the error bars indicate standard deviation (± SE). Statistical significance was analysed by Student’s t-test and *p* values less than 0.05 and 0.01 were considered as statistically significant. Asterisk above the bars (*) indicates statistically different at (p*<*0.05) and (**) indicates statistically different at (p*<*0.01) level (Supplementary Figure 5). All the transcript ID and the primer sequences used in this study is given in Supplementary Table 2.

**1.2 Quantification of diosgenin, trigonelline, and 4-hydroxyisoleucine in different tissue samples**

**1.2.1** *Sample preparation*: Fenugreek cultivar AFG-1 plants were grown as mentioned above (Supplementary Methods 1.1) and leaf, stem, and root tissues were collected from one month old plants. The dry seeds (0 hour) procured from College of Horticulture, GKVK Campus, Bangalore were soaked in distilled water and samples were collected at 12 and 24 hours. All samples were flash-frozen in liquid nitrogen, ground to a fine powder using a mortar and pestle, further dried and stored at room temperature in dark. The powdered samples were subjected to quantitative analysis using High Performance Liquid Chromatography-Photometric Diode Array (HPLC-PDA) (Shimadzu Nexera UHPLC) using the Agilent Eclipse Plus C18 column for the presence of diosgenin and trigonelline, and Liquid Chromatography-Mass Spectrometry (LC-MS) for the presence of 4-hydroxyisoleucine (4-HIL). For detecting diosgenin, 50 mg of each of the samples was weighed and 300μl of 3.5N HCl was added to the samples and vortexed. Samples were incubated at 37°C for 3 hours for acid hydrolysis and kept at 65°C overnight for complete drying. The dried samples were washed twice with H_2_O and the water wash discarded and pellet retained. 1ml of chloroform was added to the pellet and incubated at 35°C overnight at 550 rpm. Samples were centrifuged and the chloroform layer was separated. The chloroform layer was dried under vacuum and reconstituted in 300μl of 100% methanol. Each sample was vortexed, centrifuged at 14800 rpm for 5 min. Total of 10μl from the above was injected into the HPLC-PDA system for analysis. For detecting trigonelline, 10 mg of each of the tissue samples was weighed and 200 μl of 90% methanol was added to the samples and vortexed. Samples were sonicated for 2 min and centrifuged at 14000 rpm for 5 min and supernatant was separated. The supernatant was separated and centrifuged again for 5 min at 14000 rpm. 10 μl of supernatant was injected into the HPLC-PDA system for analysis. For 4-HIL detection, 50 mg of each of the samples was weighed and 200 μl of 80% methanol was added to the samples and vortexed. Samples were sonicated for 2 min and centrifuged at 14800 rpm for 5 min and supernatant was separated, from which, 40 μl was taken and 10 μl of internal standard was added to each sample. Samples were centrifuged again at 14800 rpm for 5 min and supernatant was separated. Total 8 μl of supernatant was injected into LC-MS for analysis.

**1.2.2** *Preparation for Standards*: 1 mg ml^-1^ (w/v) of standard diosgenin was prepared using methanol. Total 200 μl of the solution was dried under vacuum and reconstituted in 80 μl of 100% methanol to get a concentration of 2.5 mg ml^-1^. This was diluted serially (2-fold) in 100% methanol to get a 6-point dilution. 10 μl was dried under vacuum and reconstituted in 50 μl of 100% methanol. The standard was vortexed, centrifuged for 5 min at 14800 rpm. 10 μl of the supernatant was injected into the HPLC-PDA system for analysis. 1 mg ml^-1^ (w/v) of standard trigonelline hydrochloride was prepared in methanol. 10 μl of the solution was diluted to 990 μl of 90% methanol to get a concentration of 10 μg ml^-1^. This was diluted serially (2-fold) in 90% methanol to get a 10-point calibration curve. 10 μl of this solution was injected into the HPLC-PDA system for analysis. 25 μg ml^-1^ of standard 4-HIL and 25 μg ml^-1^ of internal standard (Leu-D10) were prepared separately from respective 1mg ml^-1^ stocks in 0.1N HCl. Standards were diluted serially in 0.1N HCl. 10 μl of the highest and lowest concentrations were taken separately and spiked into 30 μl of methanol. 10 μl of internal standard was spiked in the above two standards. 8 μl from above was injected into LC-MS for analysis.

**1.2.3** *HPLC-PDA analysis*: For quantification of diosgenin and trigonelline, Shimadzu Nexera UHPLC was used. For maximum separation of diosgenin an Agilent Eclipse Plus C_18_ column (dimensions: 5μ, 250 mm x 4.6 mm) was used with the following gradient program: 0-12 min: 95% B, 12-12.1 min: 95-100% B, 12.1-16 min: 100% B, 16-16.1 min: 100-95% B, 16.1-20 min: 95% B. The flow rate was set to 1 ml min^-1^. For maximum separation of trigonelline Phenomenex, Luna 5μ HILIC column (dimensions: 200 Å, 150 mm x 4.6 mm) was used with the following gradient program: 0-3 min: 90% B, 3-18 min: 90-20% B, 18-19 min: 20-5% B, 19-20 min: 5% B, 20-20.1 min: 5-90% B and 20.1-25 min: 90% B. The flow rate was set to 0.8 ml min^-1^. The UHPLC system is equipped with a column oven (set at 45°C), autosampler and a thermo-controller (set at 10°C). The mobile phase solvent A was water (10 mM ammonium acetate) containing 0.1% formic acid and solvent B was acetonitrile containing 0.1% formic acid. The PDA detection range was set to 190-600 nm.

**1.2.4** *LC-MS analysis:* The mass spectrometer (MS) used for the 4-HIL analysis was a Thermo Fisher- Q Exactive MS (Thermo Fisher Scientific, San Jose, CA, USA). The MS is coupled to a Dionex Ultimate3000 UHPLC system. For maximum separation, Phenomenex Luna® NH2 100 Å column (dimensions: 5μ, 150 mm x 4.6 mm) was used with the following gradient program: 0-3 min: 100-90% B, 3-4 min: 90-65% B, 4-15 min: 65-20% B, 15-17 min: 20-0% B, 17-20 min: 0% B, 20- 20.1 min: 0-100% B, 20.1-25 min: 100% B. The flow rate was set to 0.4 ml min^-1^. This UHPLC system is equipped with a column oven (set at 40°C), autosampler and a thermo-controller (set at 10°C). It uses a flow through injection mode and is equipped with a needle wash system (with acetonitrile, 0.1% formic acid) before injection to ensure 0% carry over problems. The mobile phase solvent A was water (10 mM ammonium acetate) containing 0.1% formic acid and solvent B was acetonitrile containing 0.1% formic acid. The operating conditions for the MS were as follows: spray voltage: (+) 4000V; vaporizer temperature: 320°C; sheath gas: 30 (arbitrary units); auxiliary gas: 10 (arbitrary units); acquisition mode: FSMS; scan range: 70-1000 m/z; resolution: 70000.

**2. Supplementary Data:**

The additional supplementary data is available at following link: <http://caps.ncbs.res.in/download/tfoe_data/>

**Supplementary Data 1: Annotation report:** All the transcripts (transcript IDs) are documented along with the functional annotation from various sources.

**Supplementary Data 2:** Details of validation for determining the candidate transcripts for enzymes involved in selected secondary metabolites biosynthesis pathway. The validation follows mapping of FIRs and co-clustering with the closely related species. The pathway information wherever available is also provided.

**3. Supplementary Figures:**

**Supplementary Figure 1: Functional annotation of fenugreek transcriptome.** (A) Pfam domain distribution in the ORFs. Top 20 most abundant domain families have been represented. (B) Distribution of GO term categories. The bars represent transcripts (percentage represented in log10 values). The bar plot was generated using WEGO tool (<https://wego.genomics.cn/>).

**Supplementary Figure 2: Orthogroup distribution:** Orthogroup distribution of *T. foenum-graecum* compared in a set of 39 plant species (including *T. foenum-graecum*) represented as a horizontal stacked bar graph (List of species: Supplementary Table 1). Each bar graph comprises of stacks representing the number of species sharing the total orthogroups. For each species bar, ‘1’ represents the number of orthogroups unique to that species, while ‘2’ represents the number of orthogroups shared between any of the two species among the set of 33 plants and so on till ‘33’ representing number of orthogroups found in that species shared with all the 33 plants.

**Supplementary Figure 3:** (A) The Pfam domain distribution observed in the singletons (top 15 most abundant domains have been represented). The distribution of most common GO terms in the singletons (B: Molecular function, C: Biological process, D: Cellular component).

**Supplementary Figure 4:** Clustering and alignment of enzyme- nicotinate N-methyltransferase (NNMT) involved in the biosynthesis of trigonelline with known sequences from other plants. The five functionally important residues (FIRs) in the multiple sequence alignment, considered for validation of sequence hits in fenugreek, are represented in panels A-D in blue boxes (A- Asn21, B- Tyr/Trp120, His 124, C- Thr264 (catalytic residue), D- SAM binding motif 3). The phylogenetic tree representing clustering of NNMT transcript (Tfoe_c19814_g2_i1_m7872) with *Medicago truncatula* NNMT sequence (NCBI RefSeq ID: XP_013464471.1).

**Supplementary Figure 5:** qRT-PCR validation of select transcripts in different plant parts (A) NNMT, (B) Sterol-3β-glucosyltransferase-1, (C) Sterol-3β-glucosyltransferase-2 and (D) β-glucosidase enzymes. The bars represent the qRT-PCR expression and the line represents average TPM values. Asterisk above the bars (*) indicates statistically different at (p<0.05) and (**) indicates statistically different at (p<0.01) level.

**Supplementary Figure 6:** Standard curves for diosgenin (A) and trigonelline (B) obtained through HPLC quantification.

**Supplementary Figure 7:** HPLC data for trigonelline (A-D), diosgenin (E-H) across tissues (leaf, root, stem and seed). LC data for 4-HIL leaf (I), stem (J), root (K) and from seeds (L-N: 0 hours, 12 hours and 24 hours post soaking).

**4. Supplementary Tables:**

**Supplementary Table 1:** List of 38 plants selected for the orthology analysis. The source of the proteome from the Phytozome resource is noted for each plant along with its associated citation.

| **Species** | **Phytozome12.1.2 assembly version** | **Phytozome12.1.2 annotation version** | **Associated paper citation** | **Phytozome citation** |
| --- | --- | --- | --- | --- |
| *Aquilegia coerulea* | V3.0 | V3.1 | - | Aquilegia coerulea Genome Sequencing Project, <http://phytozome.jgi.doe.gov/pz/portal.html#!info?alias=Org_Acoerulea> |
| *Arabidopsis lyrata* | V1.0 | V2.1 | Hu, Tina T., et al. "The Arabidopsis lyrata genome sequence and the basis of rapid genome size change." *Nature genetics* 43.5 (2011): 476-481. doi: 10.1038/ng.807. Epub 2011 Apr 10. PubMed PMID: 21478890; PubMed Central PMCID: PMC3083492. | - |
| *Arabidopsis thaliana* | TAIR10 | TAIR10 | Lamesch, Philippe, et al. "The Arabidopsis Information Resource (TAIR): improved gene annotation and new tools." *Nucleic acids research* 40.D1 (2012): D1202-D1210. doi: 10.1093/nar/gkr1090. Epub 2011 Dec 2. PubMed PMID: 22140109; PubMed Central PMCID: PMC3245047. | - |
| *Boechera stricta* | - | V1.2 | - | Boechera stricta v1.2, DOE-JGI, <http://phytozome.jgi.doe.gov/pz/portal.html#!info?alias=Org_Bstricta> |
| *Brachypodium distachyon* | V3.0 | V3.1 | International Brachypodium Initiative. Genome sequencing and analysis of the model grass Brachypodium distachyon. Nature. 2010 Feb 11;463(7282):763-8. doi: 10.1038/nature08747. PubMed PMID: 20148030. | - |
| *Brassica rapa* | V1.3 | V1.3 | - | Brassica rapa FPsc v1.3, DOE-JGI, <http://phytozome.jgi.doe.gov/pz/portal.html#!info?alias=Org_BrapaFPsc> |
| *Capsella grandiflora* | V1.1 | V1.1 | Slotte, Tanja, et al. "The Capsella rubella genome and the genomic consequences of rapid mating system evolution." *Nature genetics* 45.7 (2013): 831-835. doi: 10.1038/ng.2669. Epub 2013 Jun 9. PubMed PMID: 23749190. | - |
| *Capsella rubella* | V1.0 | V1.0 | Slotte, Tanja, et al. "The Capsella rubella genome and the genomic consequences of rapid mating system evolution." *Nature genetics* 45.7 (2013): 831-835. doi: 10.1038/ng.2669. Epub 2013 Jun 9. PubMed PMID: 23749190. | - |
| *Carica papaya* | V0.4 | V0.4 | Ming, Ray, et al. "The draft genome of the transgenic tropical fruit tree papaya (Carica papaya Linnaeus)." *Nature* 452.7190 (2008): 991-996. doi: 10.1038/nature06856. PubMed PMID: 18432245; PubMed Central PMCID: PMC2836516. | - |
| *Citrus clementina* | V1.0 | V1.0 | - | Haploid Clementine Genome, International Citrus Genome Consortium, 2011, <http://int-citrusgenomics.org/>, <http://phytozome.jgi.doe.gov/pz/portal.html#!info?alias=Org_Cclementina> |
| *Citrus sinensis* | V1.1 | V1.1 | - | Sweet Orange Genome Project 2010, <http://phytozome.jgi.doe.gov/pz/portal.html#!info?alias=Org_Csinensis> |
| *Cucumis sativus* | - | V1.0 | Huang, Sanwen, et al. "The genome of the cucumber, Cucumis sativus L." *Nature genetics* 41.12 (2009): 1275-1281. doi: 10.1038/ng.475. Epub 2009 Nov 1. | - |
| *Eucalyptus grandis* | V2.0 | V2.0 | - | Eucalyptus grandis Genome Project 2010, <http://phytozome.jgi.doe.gov/pz/portal.html#!info?alias=Org_Egrandis> |
| *Eutrema salsugineum* | V1.0 | V1.0 | Yang, Ruolin, et al. "The reference genome of the halophytic plant Eutrema salsugineum." *Frontiers in plant science* 4 (2013): 46. doi: 10.3389/fpls.2013.00046. eCollection 2013. PubMed PMID: 23518688; PubMed Central PMCID: PMC3604812. | Eutrema salsugineum Genome Project 2011, <http://phytozome.jgi.doe.gov/pz/portal.html#!info?alias=Org_Esalsugineum> |
| *Fragaria vesca* | - | V1.1 | Shulaev, Vladimir, et al. "The genome of woodland strawberry (Fragaria vesca)." *Nature genetics* 43.2 (2011): 109-116  . doi: 10.1038/ng.740. Epub 2010 Dec 26. PubMed PMID: 21186353; PubMed Central PMCID: PMC3326587. | - |
| *Glycine max* | V2.0 | A2.V1 | Schmutz, Jeremy, et al. "Genome sequence of the palaeopolyploid soybean." *nature* 463.7278 (2010): 178-183. doi: 10.1038/nature08670. Erratum in: Nature. 2010 May 6;465(7294):120. PubMed PMID: 20075913. | - |
| *Gossypium raimondii* | V2.1 | V2.1 | Paterson, Andrew H., et al. "Repeated polyploidization of Gossypium genomes and the evolution of spinnable cotton fibres." *Nature* 492.7429 (2012): 423-427. doi: 10.1038/nature11798. PubMed PMID: 23257886. | - |
| *Linum usitatissimum* | V1.0 | V1.0 | Wang, Zhiwen, et al. "The genome of flax (Linum usitatissimum) assembled de novo from short shotgun sequence reads." *The Plant Journal* 72.3 (2012): 461-473. doi: 10.1111/j.1365-313X.2012.05093.x. Epub 2012 Aug 14. PubMed PMID: 22757964. | - |
| *Malus domestica* | - | V1.0 | Velasco, Riccardo, et al. "The genome of the domesticated apple (Malus× domestica Borkh.)." *Nature genetics* 42.10 (2010): 833-839. doi: 10.1038/ng.654. Epub 2010 Aug 29. PubMed PMID: 20802477. | - |
| *Manihot esculenta* | V6.0 | V6.1 | Prochnik, Simon, et al. "The cassava genome: current progress, future directions." *Tropical plant biology* 5.1 (2012): 88-94. Epub 2012 Jan 5. PubMed PMID: 22523606; PubMed Central PMCID: PMC3322327. | - |
| *Medicago truncatula* | Mt4.0 | 4.0v1 | Young, Nevin D., et al. "The Medicago genome provides insight into the evolution of rhizobial symbioses." *Nature* 480.7378 (2011): 520-524. doi: 10.1038/nature10625. PubMed PMID: 22089132; PubMed Central PMCID: PMC3272368. | - |
| *Mimulus guttatus* | 4.0v1 | V2.0 | Hellsten, Uffe, et al. "Fine-scale variation in meiotic recombination in Mimulus inferred from population shotgun sequencing." *Proceedings of the National Academy of Sciences* 110.48 (2013): 19478-19482. doi: 10.1073/pnas.1319032110. Epub 2013 Nov 13. PubMed PMID: 24225854; PubMed Central PMCID: PMC3845195. | - |
| *Oryza sativa* | V7.0 | V7.0 | Ouyang, Shu, et al. "The TIGR rice genome annotation resource: improvements and new features." *Nucleic acids research* 35.suppl_1 (2007): D883-D887. Epub 2006 Dec 1. PubMed PMID: 17145706; PubMed Central PMCID: PMC1751532. | - |
| *Panicum virgatum* | V1.0 | V1.1 | - | Panicum virgatum v1.0, DOE-JGI, <http://www.phytozome.net/pvirgatum> |
| *Phaseolus vulgaris* | V2.1 | V2.1 | - | Phaseolus vulgaris v1.0, DOE-JGI and USDA-NIFA, <http://phytozome.jgi.doe.gov/pz/portal.html#!info?alias=Org_Pvulgaris> |
| *Physcomitrella patens* | V3.0 | V3.3 | Rensing, Stefan A., et al. "The Physcomitrella genome reveals evolutionary insights into the conquest of land by plants." *Science* 319.5859 (2008): 64-69. Epub 2007 Dec 13. PubMed PMID: 18079367. | Physcomitrella patens v3.0, DOE-JGI,v  <http://phytozome.jgi.doe.gov/pz/portal.html#!info?alias=Org_Ppatens> |
| *Populus trichocarpa* | V3.0 | V3.0 | Tuskan, Gerald A., et al. "The genome of black cottonwood, Populus trichocarpa (Torr. & Gray)." *science* 313.5793 (2006): 1596-1604. PubMed PMID: 16973872. | - |
| *Prunus persica* | V2.0 | V2.1 | Verde, Ignazio, et al. "The high-quality draft genome of peach (Prunus persica) identifies unique patterns of genetic diversity, domestication and genome evolution." *Nature genetics* 45.5 (2013): 487-494. doi: 10.1038/ng.2586. Epub 2013 Mar 24. PubMed PMID:23525075. | - |
| *Ricinus communis* | V0.1 | V0.1 | Chan, Agnes P., et al. "Draft genome sequence of the oilseed species Ricinus communis." *Nature biotechnology* 28.9 (2010): 951-956. doi: 10.1038/nbt.1674. Epub 2010 Aug 22. PubMed PMID: 20729833; PubMed Central PMCID: PMC2945230. | - |
| *Selaginella moellendorffii* | - | V1.0 | Banks, Jo Ann, et al. "The Selaginella genome identifies genetic changes associated with the evolution of vascular plants." *science* 332.6032 (2011): 960-963. doi: 10.1126/science.1203810. Epub 2011 May 5. PubMed PMID:21551031; PubMed Central PMCID: PMC3166216. | - |
| *Setaria italica* | V2.2 | V2.2 | Bennetzen, Jeffrey L., et al. "Reference genome sequence of the model plant Setaria." *Nature biotechnology* 30.6 (2012): 555-561. doi: 10.1038/nbt.2196.PubMed PMID: 22580951. | - |
| *Solanum lycopersicum* | ITAGv2.4 | ITAGv2.4 | Tomato Genome Consortium. The tomato genome sequence provides insights into fleshy fruit evolution. Nature. 2012 May 30;485(7400):635-41. doi: 10.1038/nature11119. PubMed PMID: 22660326; PubMed Central PMCID: PMC3378239. | - |
| *Solanum tuberosum* | V3.4 | V3.4 | Potato Genome Sequencing Consortium et al. “Genome sequence and analysis of the tuber crop potato.” *Nature* vol. 475,7355 189-95. 10 Jul. 2011, doi:10.1038/nature10158  . PubMed PMID: 21743474. | - |
| *Sorghum bicolor* | V3.0.1 | V3.1.1 | Paterson, Andrew H., et al. "The Sorghum bicolor genome and the diversification of grasses." *Nature* 457.7229 (2009): 551-556. doi: 10.1038/nature07723. PubMed PMID: 19189423. | - |
| *Theobroma cacao* | V1.1 | V1.1 | Motamayor, Juan C., et al. "The genome sequence of the most widely cultivated cacao type and its use to identify candidate genes regulating pod color." *Genome biology* 14.6 (2013): 1-25. PubMed PMID: 23731509. | - |
| *Vitis vinifera* | - | 12x? | Jaillon, Olivier, et al. "The grapevine genome sequence suggests ancestral hexaploidization in major angiosperm phyla." *nature* 449.7161 (2007): 463-7. Epub 2007 Aug 26. PubMed PMID: 17721507. | - |
| *Zea mays* | AGPv3 | AGPv3 | Schnable, Patrick S., et al. "The B73 maize genome: complexity, diversity, and dynamics." *science* 326.5956 (2009): 1112-1115. doi: 10.1126/science.1178534. Erratum in: Science. 2012 Aug 31;337(6098):1040. PubMed PMID: 19965430. | - |

**Supplementary Table 2:**

The list of transcript IDs for the enzymes involved in the biosynthesis of selected metabolites and the primer sequence used for quantification through qRT-PCR

| **Metabolite** | **Enzyme** | **Transcript ID** | **Forward Primer (5′-3′)** | **Reverse Primer (5′-3′)** |
| --- | --- | --- | --- | --- |
| Trigonelline | Nicotinate N-methyltransferase | Tfoe_c19814_g2_i1_m.7872 | ctcatgtgggtggtgacatgttc | cctgctgggagtgccttgtaac |
| Diosgenin | Sterol-3β-glucosyltransferase | Tfoe_c26467_g1_i1_m.12030 | cctccacctatcccagttgacg | gacggctctgtatcaggctcg |
|  |  | Tfoe_c33087_g1_i3_m.19456 | tacatcagaaagagttgggaccagc | ggagttggcagtggtagctcatcag |
|  | β-glucosidase | Tfoe_c120410_g1_i1_m.78951 | gcttgccattgcatgagatgctag | gactttgggtgcctagtgagtcc |
| Glyceraldehyde 3-phosphate dehydrogenase (GAPDH)  (house-keeping gene as internal control) |  | Tfoe_c23803_g1_i1_m.10206 | ctcgagaagcccgccacttatgatc | actcgttgtcataccatgagacgagc |

**Supplementary Table 3:** The tissue-wide average TPM values reflecting abundance of enzymes involved in the biosynthesis of metabolites. The transcript ID with an asterisk ‘*’ is the representative hit considered in case of multiple hits in the fenugreek transcriptome.

| **Metabolites** | **Enzymes involved** | **EC number** | **Transcript ID** | **Leaf** | **Stem** | **Root** |
| --- | --- | --- | --- | --- | --- | --- |
| **1. Alkaloids** | | | | | | |
| **Trigonelline** | Nicotinate N-methyltransferase | 2.1.1.7 | Tfoe_c19814_g2_i1_m.7872* | 123.95 | 22.33 | 10.17 |
| **Glycine-betaine** | Choline monooxygenase | 1.14.15.7 | Tfoe_c38951_g1_i2_m.30577* | 7.02 | 2.93 | 1.74 |
|  | Betaine-aldehyde dehydrogenase | 1.2.1.8 | Tfoe_c82300_g1_i1_m.72031* | 46.95 | 53.76 | 37.45 |
| **2. Saponins** | | | | | | |
| **Diosgenin** | Sterol-3β-glucosyltransferase | 2.4.1.173 | Tfoe_c26467_g1_i1_m.12030* | 19.80 | 20.50 | 14.37 |
|  |  |  | Tfoe_c33087_g1_i3_m.19456* | 14.23 | 7.39 | 5.57 |
|  | Beta Glucosidase | 3.2.1.21 | Tfoe_c120410_g1_i1_m.78951* | 8.13 | 29.43 | 27.75 |
| **3. Volatiles** | | | | | | |
| **Eugenol** | Coniferyl alcohol acetyltransferase | 2.3.1.224 | Tfoe_c37673_g1_i1_m.27189* | 0.02 | 3.04 | 269.36 |
|  |  |  | Tfoe_c102741_g1_i1_m.76275 | 1.11 | 26.52 | 0.509 |
|  | Eugenol synthase/ Isoeugenol synthase/ | 1.1.1.318/1.1.1.319 | Tfoe_c34861_g1_i1_m.21897* | 0.91 | 0.34 | 76.39 |
|  |  |  | Tfoe_c34861_g1_i2_m.21898 | 0.04 | 1.77 | 4.66 |
| **Linalool** | Farnesyl-pyrophosphate synthase | 2.5.1.1 | Tfoe_c5299_g1_i1_m.1972* | 30.00 | 35.38 | 122.75 |
|  | (3S)-linalool synthase | 4.2.3.25 | Tfoe_c11093_g1_i1_m.4195* | 50.83 | 0.17 | 0 |
|  |  |  | Tfoe_c11093_g1_i2_m.4196 | 43.90 | 0.21 | 0 |
|  | (3R)-linalool synthase | 4.2.3.26 | Tfoe_c44084_g4_i2_m.55446* | 285.97 | 5.21 | 0.14 |
| **4. Flavonoids** | | | | | | |
| **Quercetin** | Coumaroyl CoA ligase | 6.2.1.12 | Tfoe_c63771_g1_i1_m.68667* | 4.74 | 49.71 | 514.02 |
|  | Chalcone synthase | 2.3.1.74 | Tfoe_c11097_g1_i1_m.4202 | 957.26 | 6.86 | 0.27 |
|  |  |  | Tfoe_c39596_g1_i1_m.32698 | 2.12 | 8.15 | 572.28 |
|  |  |  | Tfoe_c35588_g1_i1_m.23483 | 0.68 | 0 | 1.03 |
|  |  |  | Tfoe_c45060_g1_i2_m.63784* | 65.30 | 15.81 | 995.01 |
|  | Chalcone flavone isomerase | 5.5.1.6 | Tfoe_c46133_g1_i1_m.65924* | 4.78 | 87.02 | 492.99 |
|  |  |  | Tfoe_c25881_g1_i1_m.11291 | 24.67 | 20.19 | 209.70 |
|  |  |  | Tfoe_c25881_g1_i2_m.11294 | 24.26 | 1.11 | 21.81 |
|  | Flavanol-3-hydoxylase/Flavanol synthase | 1.14.11.9/1.14.20.6 | Tfoe_c82862_g1_i1_m.72503* | 271.29 | 26.80 | 104.49 |
|  | Tricin synthase | 2.1.1.175 | Tfoe_c11294_g1_i1_m.4455* | 41.27 | 609.17 | 692.68 |
|  |  |  | Tfoe_c14575_g1_i2_m.5867 | 0.12 | 4.23 | 39.32 |
|  |  |  | Tfoe_c1782_g1_i1_m.777 | 1.68 | 7.24 | 5.21 |
|  |  |  | Tfoe_c31331_g1_i1_m.17018 | 67.23 | 117.95 | 137.79 |
|  |  |  | Tfoe_c40564_g1_i2_m.36008 | 4.44 | 0.83 | 0.46 |
|  |  |  | Tfoe_c40564_g1_i6_m.36010 | 0.75 | 0.27 | 0.046 |
|  | Flavanol-3-monooxygenase | 1.14.14.82 | Tfoe_c45104_g3_i2_m.64189* | 1.48 | 46.28 | 0.58 |
|  |  |  | Tfoe_c45104_g3_i1_m.64187 | 9.32 | 0.42 | 0.15 |
|  |  |  | Tfoe_c73221_g1_i1_m.71120 | 0.142883 | 8.81171 | 0.0488886 |
|  |  |  | Tfoe_c2268_g2_i1_m.1035* | 24.4326 | 68.2354 | 84.6301 |
| **Vitexin/Isovitexin** | Naringenin 2-hydroxylase | 1.14.14.162 | Tfoe_c121119_g1_i1_m.79529* | 0 | 0 | 1.65 |
|  |  |  | Tfoe_c42265_g1_i1_m.43467* | 0.16 | 14.42 | 0.73 |
|  |  |  | Tfoe_c42265_g1_i2_m.43468 | 4.28 | 6.16 | 2.02 |
|  |  |  | Tfoe_c6548_g1_i1_m.2589 | 0 | 0.78 | 4.60 |
|  | C-glucosyltransferase | 2.4.1.360 | Tfoe_c126924_g1_i1_m.81066 | 0.05 | 0.31 | 0.02 |
|  |  |  | Tfoe_c1387_g1_i1_m.558 | 6.34 | 5.99 | 3.32 |
|  |  |  | Tfoe_c23925_g1_i1_m.10351 | 139.34 | 79.00 | 20.54 |
|  |  |  | Tfoe_c23925_g1_i2_m.10352 | 0.02 | 0.39 | 23.93 |
|  |  |  | Tfoe_c41040_g4_i1_m.37580 | 0.01 | 6.53 | 101.06 |
|  |  |  | Tfoe_c41678_g1_i1_m.40629 | 23.20 | 10.05 | 16.65 |
|  |  |  | Tfoe_c41952_g1_i1_m.41839 | 6.45632 | 2.0374 | 1.96073 |
|  |  |  | Tfoe_c41952_g1_i2_m.41840 | 3.08637 | 2.51711 | 1.75407 |
|  |  |  | Tfoe_c41952_g1_i3_m.41841 | 0.000232313 | 0.129057 | 16.1016 |
|  |  |  | Tfoe_c44021_g1_i3_m.54818 | 3.91979 | 2.29111 | 18.4141 |
|  |  |  | Tfoe_c44021_g1_i2_m.54817 | 45.5956 | 8.83118 | 12.4081 |
|  |  |  | Tfoe_c44021_g1_i3_m.54818 | 3.91979 | 2.29111 | 18.4141 |
|  |  |  | Tfoe_c44021_g1_i4_m.54819 | 8.72292 | 8.24935 | 10.8616 |
| **Rutin** | Flavonol-3-o-glucosyltransferase | 2.4.1.91 | Tfoe_c120532_g1_i1_m.79077* | 31.8749 | 5.95413 | 0.39829 |
|  |  |  | Tfoe_c138057_g1_i1_m.82271 | 3.24827 | 20.4489 | 70.1211 |
|  |  |  | Tfoe_c42342_g1_i1_m.43838 | 34.2924 | 10.5106 | 17.842 |
|  |  |  | Tfoe_c41977_g1_i1_m.42043 | 109.42 | 45.0806 | 68.9572 |
|  |  |  | Tfoe_c43221_g1_i1_m.49071 | 2.16 | 4.90 | 131.71 |
|  |  |  | Tfoe_c43641_g4_i1_m.52214 | 6.99 | 1.51 | 0.11 |
|  |  |  | Tfoe_c1171_g1_i1_m.434 | 126.83 | 4.18 | 7.53 |
|  | Flavonol-3-o-glucoside rhamnosyltransferase | 2.4. 1.15 | Tfoe_c121323_g1_i1_m.79650* | 96.82 | 2.74 | 0.18 |

**Supplementary Table 4: The quantification of secondary metabolites.** Trigonelline and diosgenin were quantified using HPLC from leaf, stem, root and seed tissue samples. 4-Hydroxyisoleucine was quantified using LC-MS from the seed samples soaked at (0, 12 and 24 hours).

| **Metabolite** | **Tissue** | **Concentration (μg of metabolite/mg of tissue)** |
| --- | --- | --- |
| **Trigonelline** | Leaf | 4.715 |
|  | Stem | 0.51 |
|  | Root | 0.202 |
|  | Seed | 2.698 |
| **Diosgenin** | Leaf | 0.614 |
|  | Stem | 0.738 |
|  | Root | 1.036 |
|  | Seed | 1.56 |
| **4-Hydroxyisoleucine** | Leaf | 77.48 |
|  | Stem | 50.99 |
|  | Root | 13.24 |
|  | Seed (0 hour) | 84.77 |
|  | Seed (12 hours) | 15.46 |
|  | Seed (24 hours) | 7.11 |

**References:**

1. Sajeevan, R. S., Shivanna, M. B. & Nataraja, K. N. An Efficient Protocol for Total RNA Isolation from Healthy and Stressed Tissues of Mulberry (Morus sp.) and Other Species. *American Journal of Plant Sciences* (2014) doi:10.4236/ajps.2014.513221.

2. Schmittgen, T. D. & Livak, K. J. Analyzing real-time PCR data by the comparative CT method. *Nature Protocols* (2008) doi:10.1038/nprot.2008.73.

3. Pasha, S. N. *et al.* The transcriptome enables the identification of candidate genes behind medicinal value of Drumstick tree (Moringa oleifera). *Genomics* (2020) doi:10.1016/j.ygeno.2019.04.014.
